# Valorization of cocoa pod side streams improves nutritional and sustainability aspects of chocolate

**DOI:** 10.1038/s43016-024-00967-2

**Published:** 2024-05-21

**Authors:** Kim Mishra, Ashley Green, Johannes Burkard, Irina Gubler, Roberta Borradori, Lucas Kohler, Johannes Meuli, Ursina Krähenmann, Jotam Bergfreund, Armin Siegrist, Maria Schnyder, Alexander Mathys, Peter Fischer, Erich J. Windhab

**Affiliations:** 1 Institute of Food, Nutrition and Health, ETH Zürich, Zürich, Switzerland; 2 Max Felchlin AG, Ibach-Schwyz, Switzerland; 3 Koa Switzerland AG, Zürich, Switzerland

**Keywords:** Rheology, Environmental impact, Obesity, Chemical engineering

## Abstract

Chocolate production faces nutritional, environmental and socio-economic challenges present in the conventional cocoa value chain. Here we developed an approach that addresses these challenges by repurposing the often-discarded pectin-rich cocoa pod endocarp and converting it into a gel. This is done using cocoa pulp juice concentrate to replace traditional sugar from sugar beets. Although swelling of fibres, proteins and starches can limit gel incorporation, our proposed chocolate formulation contains up to 20 wt% gel. It also has comparable sweet taste as traditional chocolate while offering improved nutritional value with higher fibre and reduced saturated fatty acid content. A cradle-to-factory life cycle assessment shows that large-scale production of this chocolate could reduce land use and global warming potential compared with average European dark chocolate production. The process also provides opportunities for diversification of farmers’ income and technology transfer, offering potential socio-economic benefits for cocoa-producing regions.

## Abstract (web summary)

The conventional cocoa value chain has important environmental, nutritional and socio-economic implications. This study presents a chocolate formulation that combines the cocoa pod endocarp and pulp juice to create a sweetening gel that replaces refined sugar, offering improved nutritional value and reduced environmental impact while also contributing to income diversification for smallholder farmers.

## Main

**Fig. 1:**
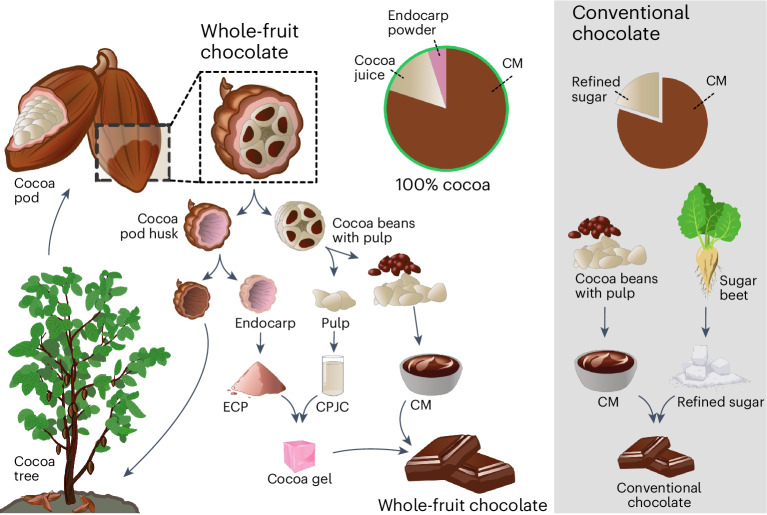
Whole-fruit and conventional chocolate manufacturing processes. Left: the whole-fruit chocolate manufacturing process, showcasing the use of the cocoa pod husk endocarp and the CPJC together with CM. The endocarp and the juice concentrate form a gel, serving as a sweetening agent and improving the nutritional profile. Right: the conventional chocolate process characterized by the utilization of refined sugar from sugar beet together with CM. CM production, chocolate moulding and cooling is done in the same way for both processes.

Despite its universal appeal, chocolate’s implications for health, socio-economics and environmental sustainability generate ongoing debate^1–4^. The average sugar content of chocolate confectionery products in the United Kingdom in 2017 was 47.3 g per 100 g and the energy density was 527 kcal per 100 g, favouring type 2 diabetes mellitus progression in the young and obese^5–8^. Furthermore, the high amount of saturated fatty acids (SFAs) in chocolate confectionery is associated with low-grade inflammation and cardiovascular disease^9^. Recent strategies to decrease SFA or increase unsaturated fatty acid intake and reduce the energy density of chocolate confectioneries involve air inclusion, additive manufacturing, and substitution of cocoa butter (CB) with oleogels or fish oil^10–14^. For a holistic outlook, such improvements in the nutritional profiles of chocolate need to be coupled with environmental impact reductions. Globally, smallholder farmers in high-biodiversity regions produce roughly 70% of cocoa, with many cocoa-producing countries relying heavily on the sector for employment, social services and foreign exchange^15–17^. Farmers face trade-offs between productivity, environmental impact and socio-economic sustainability^16^. As the world races towards a more circular and sustainable economy, innovative technologies in the food sector are required. Food utilization needs to be maximized so that associated environmental burdens can be reduced. This is particularly important for foods with higher environmental impacts, such as cocoa products. Impacts ranging from 1.25 to over 46.7 kg CO_2_ equivalents per kg of dark chocolate are reported^18–20^. The majority of these emissions arise during the cultivation of cocoa. Due to the relatively low yield (10%) of dry beans per cocoa pod^21,22^, land requirements and thus greenhouse gas emissions from the transformation of native tropical vegetation are high^23^. Therefore, an increased utilization of other parts of the cocoa pod, such as the pulp and cocoa pod husk, could not only contribute to income diversification among farmers but also reduce major environmental impacts during the cultivation phase. Ref. ^24^ showed that partial removal of pulp juice did not affect bean fermentation and could be used elsewhere^25^. Residual cocoa pod husks, which constitute up to 75% of the cocoa pod, are still discarded or used as fertilizer, potentially harbouring pests and diseases instead of being used as food^26,27^. We have developed a chocolate production process that improves the nutritional value of chocolate, environmental sustainability and income diversification of smallholder farmers (Fig. 1). Our process uses solely cocoa pod components in the chocolate. The endocarp is extracted, dried and milled to a powder. The cocoa bean pulp is harvested and pressed for its juice, with subsequent concentration. The cocoa pulp juice concentrate (CPJC) and the endocarp powder (ECP) are mixed and heated to a gel that is added to cocoa mass (CM) from cocoa beans.

## Results and discussion

### Manufacture and characterization of sweetening gels

**Fig. 2:**
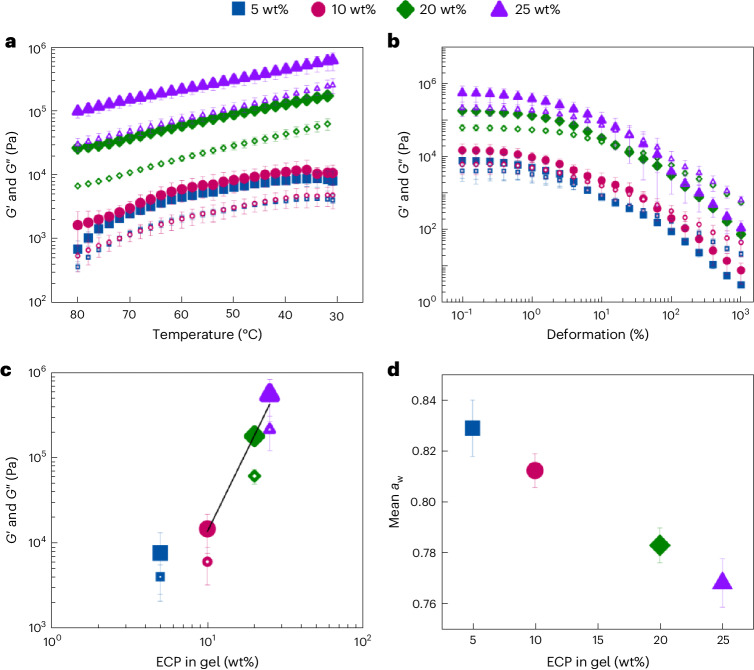
Relationship between ECP content and the sweetening gel’s material properties. **a**, *G*′ (closed symbols) and *G*″ (open symbols) of ECP gels at a pH of 3.2 as a function of temperature during cooling from 80 °C to 31 °C. **b**, *G*′ (closed symbols) and *G*″ (open symbols) of ECP gels as a function of deformation at 31 °C. **c**, *G*′ (closed symbols) and *G*″ (open symbols) of ECP gels as a function of ECP concentration derived from the amplitude sweep. **d**, *a*_w_ of ECP gels as a function of ECP concentration measured at 25 °C. All error bars correspond to the s.d. of the mean from triplicate measurements. Source data

Introducing water into CM leads to detrimental effects such as thickening and phase inversion^28^. However, using a gelling agent such as pectin to restrict water from interacting with the CM matrix can prevent these detrimental effects. The high ECP pectin content (Supplementary Table 1) is well suited for the gelation of aqueous phases high in sugars and at low pH, such as CPJC^29,30^. Different ECP concentrations were introduced into an artificial sugar solution mimicking the CPJC sugar composition to quantify ECP gelling properties (Supplementary Table 2). This solution was used to exclude any artefacts from pectins present in the CPJC. We first tested the evolution of the storage modulus (*G*′) and loss modulus (*G*″) on cooling of the gels from 80 °C to 31 °C (Fig. 2a). The endpoint of 31 °C was chosen to replicate the mixing temperatures of the gel into the pretempered CM. At all ECP concentrations tested, we observe a monotonic *G*′ and *G*″ increase with a constant slope as the temperature decreases, and a larger *G*′ than *G*″. The difference between 5 wt% and 10 wt% ECP is small compared with 20 wt% and 25 wt% ECP gels with notably higher *G*′ and *G*″. Looking at the amplitude sweeps after gelation, the linear viscoelastic regime is apparent up to a deformation of *γ* = 0.5%, and the crossover of *G*′ and *G*″ occurs close to *γ* = 10% for all tested ECP concentrations (Fig. 2b). The scaling behaviour of *G*′ and *G*″ as a function of the ECP concentration is linked to the pectin gelling mechanism (Fig. 2c). The pectin concentration of the ECP is 12–14 wt% (Supplementary Table 1), leading to effective pectin concentrations of 0.65–3.25 wt% in the ECP gels. Starting at an effective pectin concentration of 1.3 wt%, power-law dependency for both *G*′ and *G*″ is apparent with a slope of 4.6 (Fig. 2c). In addition, the water activity (*a*_w_) as a function of ECP concentration shows a linear decrease from 0.83 to 0.77 (Fig. 2d). These results suggest that gel formation occurs at all tested ECP concentrations. Ref. ^31^ reported gel formation of high-methoxyl pectin at 0.5 wt%, confirming our results. Furthermore, the slope of *G*′ and *G*″ as a function of temperature in the log–lin plot is similar for all investigated ECP concentrations. Similar slopes have been reported^32^ for pure pectin solutions, confirming that the gelation of pectin is driving the increase in *G*′ and *G*″. As evident from Supplementary Table 1, cellulose, hemicellulose and lignin are also present in the ECP. Although they are poorly soluble in acidic aqueous environments, they will increase *G*′ and *G*″ due to their presence as solid particles. The power-law dependency between *G*′ and *G*″ and the ECP concentration (Fig. 2c) have been confirmed by previous studies^33,34^. The linear dependency of the *a*_w_ as a function of ECP concentration is partially in accordance with previous studies, which report an S-type sorption isotherm with a linear regime at moderate water contents^35,36^.

### Qualitative colloidal stability

**Fig. 3:**
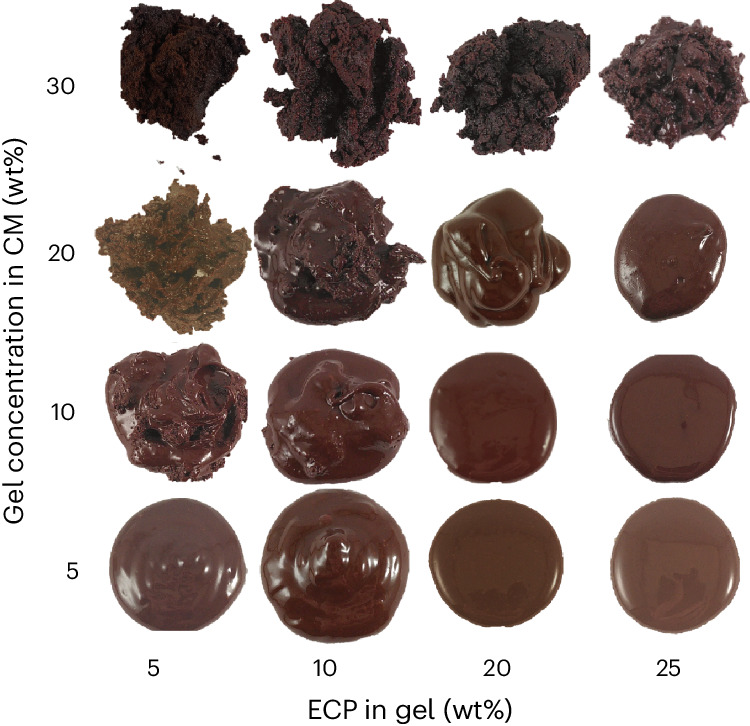
Sweetening gel addition in chocolate and related thickening effects. Images of whole-fruit chocolate formulations after kneading at 31 °C and subsequent heating to 50 °C. The ECP concentration in the sweetening gel and the added gel concentrations into the CM are shown on the *x* and *y* axis, respectively. Source data

Whole-fruit chocolate formulations were made by mixing ECP gels and CM in a kneader at 31 °C. The interaction of the free water in the ECP gels with the CM matrix determines the colloidal stability of the chocolate. Increasing the gel concentration in the CM from 5 wt% to 30 wt% decreases the gloss of the chocolate samples and induces tip and crumbly paste formation. However, this effect is much more pronounced at low concentrations of ECP in the gel. With 25 wt% ECP in the gel, only the addition of 30 wt% gel makes the sample crumbly (Fig. 3). The transition from glossy, flat drops to crumbly pastes is due to the swelling of CM matrix biopolymers on introduction of the gels. The *a*_w_ of the gels is higher compared with the biopolymers present in the CM. Hence, an equilibrium spreading of the water present in the gels throughout the CM occurs. At low gel additions and high ECP concentrations, the little available water is immobilized in the capillaries of the protein and fibre particles present in the CM. As dispersed particles do not substantially change size during the wetting of their capillaries, little rheological effects are observed. With higher gel additions, the available water leads to swelling of the dispersed biopolymer particles in the CM, thereby causing slight tip formation. At high gel additions, the swollen biopolymer particles in the CM form interparticle capillary bridges, leading to the formation of a strong network. Adding even more gel eventually leads to a phase inversion, shown by the loss of glossiness and a crumbly texture.

### Quantitative colloidal stability

**Fig. 4:**
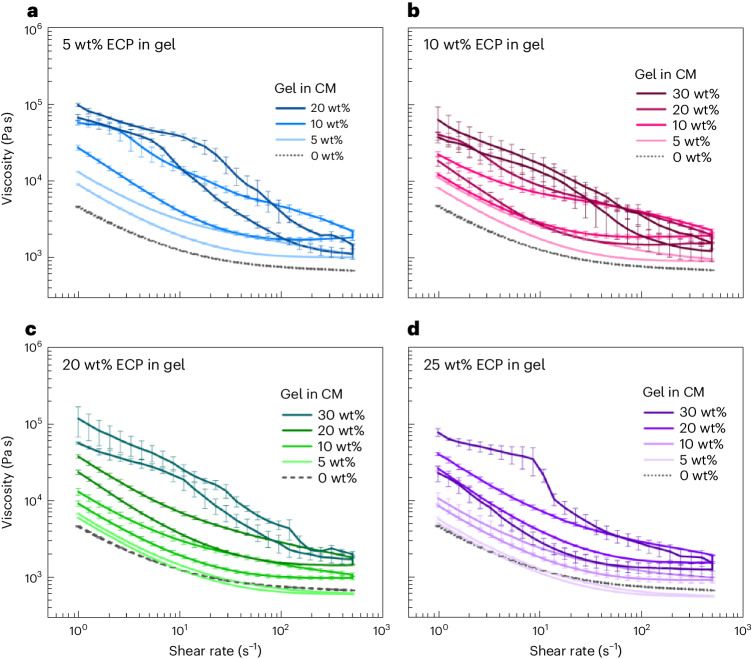
Chocolate viscosity as the main indicator for thickening and network formation effects. **a**–**d**, The viscosity as a function of shear rate for whole-fruit chocolate formulations with added sweetening gels containing 5 wt% (**a**), 10 wt% (**b**), 20 wt% (**c**) and 25 wt% (**d**) ECP. The 30 wt% is not displayed in **a**, because the sample was too solidified and not suitable for measurement. Sweetening gel concentrations of 0 wt%, 5 wt%, 10 wt%, 20 wt% and 30 wt% are shown. An upwards (top curve, the curve with higher viscosity) and downwards (bottom curve, the curve with lower viscosity) shear rate ramp at 50 °C was performed. All error bars correspond to the s.d. of the mean from triplicate measurements. Source data

**Fig. 5:**
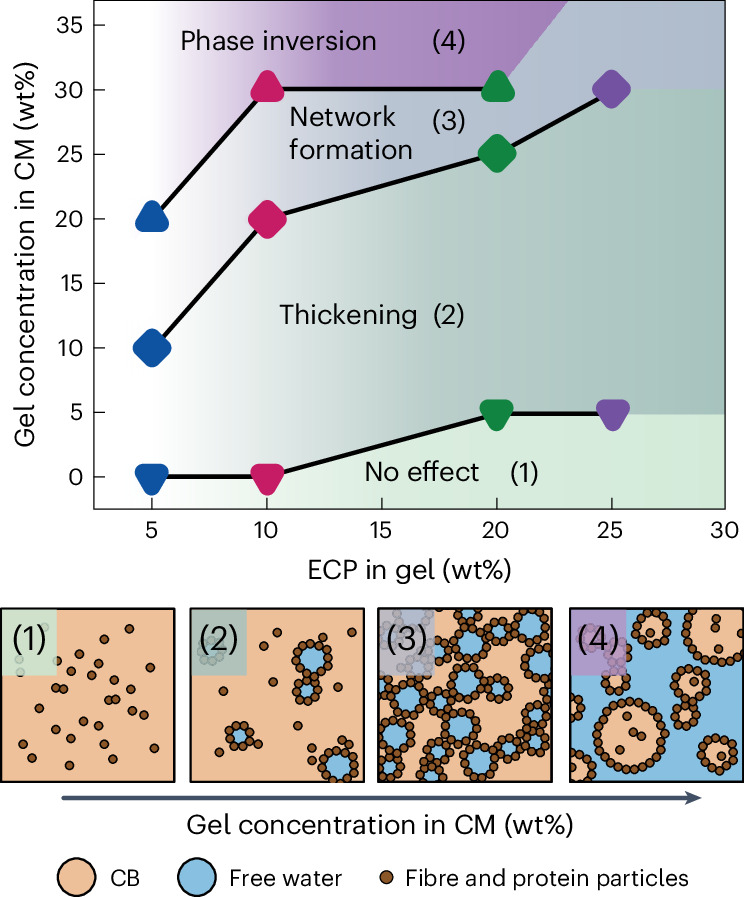
Interplay between water immobilization in the sweetening gels and chocolate thickening. State diagram of the whole-fruit chocolate formulation system indicating the (1) state of no effect, (2) thickening state, (3) network formation state and (4) phase inversion. States 1 and 2 represent workable chocolate. States 3 and 4 represent unworkable chocolate. Source data

To confirm these qualitative results, the rheology of the whole-fruit chocolate formulations after kneading was investigated further by measuring the viscosity as a function of shear rate. We show that the viscosity of the reference CM (0 wt%) is characterized by a shear thinning behaviour followed by a constant viscosity at high shear rates known as the second Newtonian plateau (Fig. 4a–d)^37^. The shear thinning results from disperse fibre and protein particles orienting themselves in the flow field. The reference CM shows identical viscosities for the upwards and downwards shear ramp, indicating the absence of thixotropy during the investigated timescales^38^. Shear thinning and thixotropic effects due to orientation and deaggregation of fat crystals does not occur because the measurement temperature is above the melting temperature of CB^39,40^. At 5 wt% ECP in the gel, adding 5 wt% gel to the CM leads to an upwards ramp with increased viscosity and the absence of a second Newtonian plateau compared with pure CM, indicating thickening effects (Fig. 4a). The downwards ramp shows decreased viscosity compared with the upwards ramp (hysteresis), indicating thixotropic behaviour. Adding 10 wt% gel to the CM leads to a first Newtonian plateau in the upwards ramp, indicating network formation such as capillary bridges^41^. The subsequent strong shear thinning behaviour without a second Newtonian plateau is due to the deaggregation of the network and alignment of dispersed particles in the flow field. The downwards ramp shows a second Newtonian plateau and lowered viscosity compared with the upwards ramp showing thixotropy. Adding 20 wt% gel to the CM leads to pronounced first Newtonian plateaus in the upwards and the downwards ramp, indicating irreversible network formation due to phase inversion^42^. Increasing the ECP concentration in the gel to 10 wt% (Fig. 4b) shows almost identical viscosity curves compared with 5 wt% ECP (Fig. 4a). At 20 wt% ECP in the gel, no hysteresis at 5 wt% gel addition is observed, indicating the absence of thickening and thixotropic effects (Fig. 4c). Gel additions of 10 wt% and 20 wt% show hysteresis and therefore thickening thixotropy. At 30 wt% gel addition, 2 first Newtonian plateaus confirm phase inversion. A 25 wt% ECP in the gel shows an identical development of the viscosity curves as 20 wt% ECP, the only difference being the absence of a first Newtonian plateau in the down ramp at 30 wt% gel addition (Fig. 4d). The quantitative and qualitative findings on the colloidal stability of the whole-fruit chocolate formulations are visualized in a state diagram (Fig. 5). With increasing ECP concentrations in the gel, higher gel additions are required to induce thickening, network formation and phase inversion of the system. In particular, above 10 wt% ECP in the gel, a substantially higher amount of gel is required to induce network formation. The state diagram shows the ECP gel concentration at which chocolate processing is still feasible. Below the formation of a network, the whole-fruit chocolate formulations are still suitable for tempering and moulding. Adding 20 wt% gel containing 20 wt% or 25 wt% ECP in the gel results in the highest sugar and fibre addition to the whole-fruit chocolate formulation with minimal effect on rheology.

### Sweetness perception

**Fig. 6:**
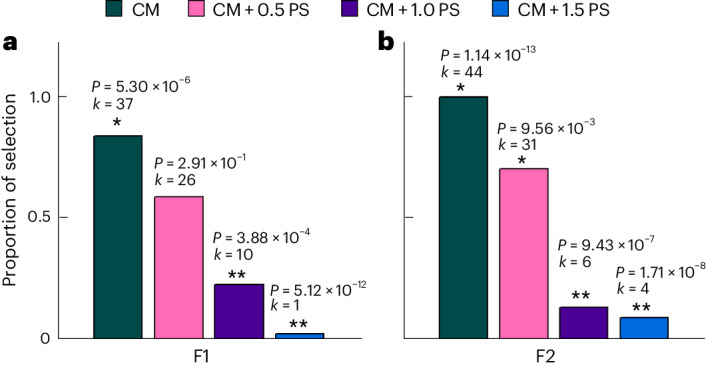
Sweetness perception of the whole-fruit chocolate formulations based on their CB content. **a**,**b**, The fraction of panellists’ choices rating the whole-fruit chocolate formulation F1 (**a**) or F2 (**b**) sweeter than pure CM or conventional dark chocolate at half (CM + 0.5 PS), equal (CM + 1 PS) or one and a half (CM + 1.5 PS) the sugar content of F1 and F2. On the basis of the ratio of the number of selected samples (*k*) to the total number of evaluations (*n* = 44), *P* values were calculated with a bidirectional binomial test with the expected proportion of success *µ* = 0.5. One asterisk (*) refers to F1 or F2 being significantly sweeter and two asterisks (**) refers to the opposite. F1 was not adjusted for CB content, whereas F2 was adjusted to the CB content of the corresponding dark chocolate (CM + 1 PS) formulation. Source data

It remains unclear whether the sweetness sensation induced by the ECP gel is comparable to the powdered sugar (PS) typically used for conventional dark chocolate. To assess the sweetness sensation of both the ECP gel and PS dispersed in the CM matrix, a sensory evaluation was performed by a two-alternative forced choice (2-AFC) test comparing the whole-fruit chocolate formulation with sweetening gels with conventional dark chocolate formulations with PS. The calculated sugar content of the whole-fruit chocolate formulation with 20 wt% gel containing 25 wt% ECP in 75 wt% CPJC was 9.6 wt%. Hence the concentration of PS in the conventional dark chocolate was set to half (CM + 0.5 PS), equal (CM + 1 PS), and one and a half (CM + 1.5 PS) the concentration introduced by the gel. To account for the difference in CB between the whole-fruit chocolate formulation and the conventional dark chocolate formulations, two different whole-fruit formulations were used (F1 and F2; shown in Supplementary Table 3). Both formulations F1 and F2 have the same sugar content (wt%) as the conventional dark chocolate sweetened with 9.6 wt% PS (CM + 1 PS). The CB content of F1 is lower than conventional dark chocolate, whereas that of F2 is matched to that of conventional dark chocolate. The panellists perceived the unsweetened CM as significantly less sweet than F1 (Fig. 6a). No significant difference in sweetness between CM + 0.5 PS and F1 was observed. The samples CM + 1 PS and CM + 1.5 PS were perceived as significantly sweeter than F1. Several comments on strong acidity and bitterness were given despite the request to ignore such effects. A negative correlation between sweetness sensation and bitterness in cocoa-based systems has been shown^43^. Nevertheless, F1 was not differentiable in sweetness perception compared with CM + 0.5 PS. An increment in fat content from 44 wt% to 49.4 wt% resulted in F2 being significantly sweeter than CM + 0.5 PS. A higher fat content might have resulted in a bitterness suppression, as described by ref. ^44^. Compared with CM + 1 PS and CM + 1.5 PS, F2 was perceived as significantly less sweet. A reason for the reduced sweetness perception of F1 and F2 compared with conventional dark chocolate formulations is found in the mobility of sugar molecules. Once introduced into the mouth, migration of sugar molecules from the sweetening gels into the saliva requires a solvent exchange. Both the CPJC and the saliva are relatively good solvents, decreasing the rate of solvent exchange. PS in conventional dark chocolate is present in its crystalline state and readily dissolves into saliva. Furthermore, gel particle size is increased compared with the finely ground PS, thereby reducing overall particle surface area and leading to even lower sugar release rates. The sensory study shows that the whole-fruit chocolate formulations are perceived as comparably sweet as conventional dark chocolate.

### Life cycle assessment

**Fig. 7:**
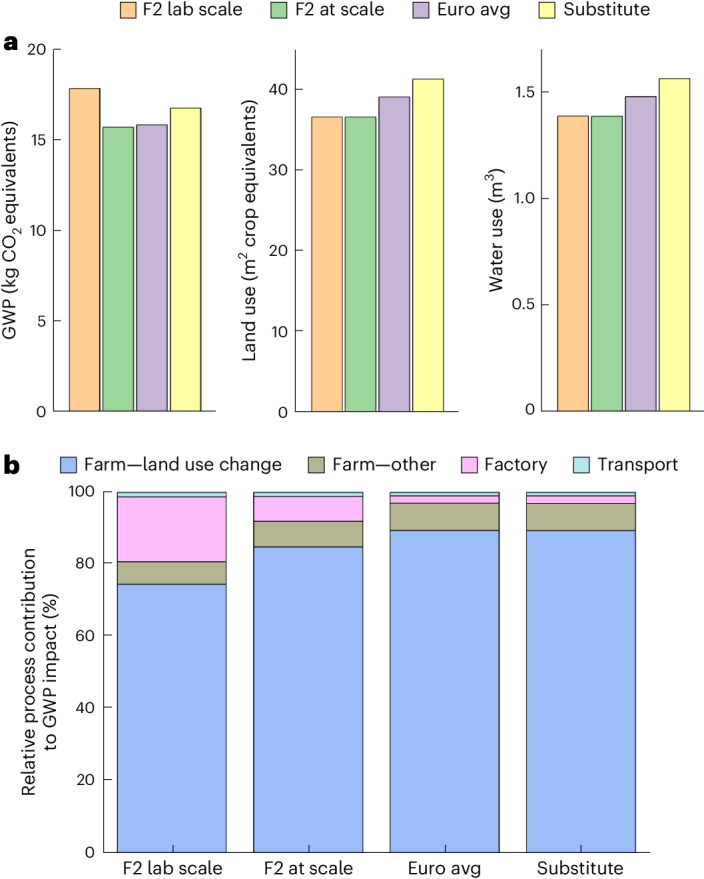
Sustainability analysis comparing the different chocolates. **a**,**b**, Environmental impact for GWP (kg CO_2_ equivalents), land use (m^2^ area crop equivalents), and water use (m^3^) (**a**) and relative process contributions (**b**) for 1 kg of F2 lab-scale, F2 at-scale, Euro avg and substitute chocolate. Farm—land-use change refers to impacts due to land-use change, farm—other refers to primary product cultivation and associated impacts excluding land-use change impacts, factory refers to processing of all primary products into intermediate and final products, and transport refers to the transport of products used in chocolate production. Source data

After the sensory test, we completed a life cycle assessment (LCA) using a functional unit of 1 kg of the product likely to be accepted by consumers based on the sweetness criterion (that is, F2). The LCA of the whole-fruit chocolate F2 was performed accounting for the actual laboratory-scale process (F2 lab scale) used to produce the sample chocolate and for a hypothetical at-scale-produced chocolate (F2 at scale). F2 lab scale and F2 at scale were compared with a conventional dark chocolate according to the European average (Euro avg) and a dark chocolate substituted with the same CM content as F2 (substitute). The formulations are shown in Supplementary Table 4. F2 lab scale shows a higher global warming potential (GWP) impact compared with the Euro avg and substitute (Fig. 7a). F2 at scale shows a lower GWP impact compared with the substitute and scores very similar to the Euro avg. The land and water use for the F2 lab scale and F2 at scale are the same because the at-scale scenario focused on energy use and land use is independent of the factory processes. The F2 lab scale shows the lowest land use compared with the Euro avg and substitute; the substitute scores highest for land use. Water use of the F2 lab scale is lower compared with the Euro avg and substitute. The main driver for the reduced GWP impact of the F2 at scale is the increased drying efficiency of the endocarp at scale compared with the inefficient gas oven drying in the laboratory. As the biggest impacts for land use occur during the cocoa pod cultivation stage, F2 outperforms the Euro avg and substitute by enhancing pod utilization. We next assessed the relative impacts of the group stages, namely, farm—land use change, farm—other, factory and transport (Fig. 7b). Land-use change from farm activities comprise over 70% of environmental impacts for all chocolates, whereas the rest of the farm activities contribute between 6.3% and 7.6%. The factory stage has variable impacts across chocolates, with higher values (17.8%) for the F2 lab scale and lower values for the F2 at scale (6.9%), Euro avg (2%) and substitute (2.1%). Transport impacts were low at around 1% for all chocolates. Overall F2 uses less cocoa beans by weight, which contributes to lower farm impacts. This reduced cocoa bean use is not compensated by other primary product use because the F2 uses more of the cocoa pod (that is, it uses components that would otherwise be considered waste). However, the F2 chocolate involves more processing than the Euro avg and substitute chocolate because the cocoa pulp juice and endocarp undergo several steps before being combined into the sweetening gel, contributing to more factory impacts. The majority of factory impacts is due to the ECP production. Environmental impacts associated with ECP production could be partially reduced through alternative drying methods, such as microwave drying. Moreover, increasing the amount of solar energy used by shifting away from diesel and grid energy could further reduce impacts in the factory stage. Future studies with more empirical data should conduct uncertainty analyses for whole-fruit and conventional chocolate and assess the statistical significance of their differences. In the future, F2 holds potential to be relatively more sustainable than conventional chocolate, assuming hotspot reductions (for example, drying) for ECP production are successful. We evaluated a product for which processing is completed at the laboratory scale. Once processing is optimized at scale, environmental impacts should be assessed against various dark chocolate formulations to determine comparative sustainability with updated processing parameters reflective of industrial production.

## Conclusion

Although the high sugar and SFA content of chocolate confectionery and associated adverse effects on public health are well known, awareness of chocolate’s potentially high GWP is low^18–20^. Consequently, there is an urgent need to transform the cocoa value chain addressing the nutritional and environmental problems while respecting the socio-economic boundary conditions. As shown, alternative chocolate value chains can help mitigate these issues that are inherent in the traditional cocoa value chain. Our production technique efficiently exploits cocoa pod biomass by combining the endocarp and part of the pulp juice to create sweetening gels. Empirical evidence shows the effectiveness of these gels in reducing thickening effects when combined with CM. The sweetening power of the gel is found to surpass half its weight in PS equivalents. Environmental impacts are difficult to compare across studies due to differences in system boundaries, impact assessment methods and methodological choice in how certain factors are evaluated. The geographical source of cocoa beans, chocolate product formulation and the choice of agricultural practices applied make comparisons across studies even harder. Nevertheless, the majority of impacts occur at the farm level^18–20^. Despite the extra processing needed to produce the CPJC and ECP, this chocolate formulation proves, on average, to be more environmentally friendly than its conventionally crafted counterparts. Moreover, it uses more of the cocoa crop, avoiding food loss and waste, which is of increasing concern as environmental degradation worsens. Diversified income streams for smallholder farmers and a balanced nutritional profile with increased fibre and reduced SFA content offer additional benefits over average European dark chocolate. Overall the development of whole-fruit chocolate formulations represent a promising example of how technology, nutrition, environmental impact and smallholder farmer income diversification can work together to improve the entire cocoa value chain.

## Methods

### CM

The CM was supplied by Max Felchlin. Cocoa beans from Suhum (Ghana) of the Forastero cultivar were roasted, winnowed, ground and sterilized, yielding the CM. Total fat content was 55 wt% and moisture content was <1 wt%. The composition of the mass is shown in Supplementary Table 5.

### ECP

The endocarp was provided by Koa Switzerland. It was extracted manually from cocoa pod husk harvested in Assin Akrofuom (Ghana) during June 2020. The endocarp was manually extracted from the cocoa pod husk and dried in a gas oven at 80 °C for 18 h. The dried endocarp was premilled in a roller mill and sent to Martin Bauer Group for fine milling. Fibre analysis was done according to Supplementary Method 1. The moisture content, fibre composition and particle size distribution of the resulting ECP are shown in Supplementary Table 1 and Supplementary Fig. 1.

### Artificial sugar solution

To mimic the CPJC sugar composition without residual pectin moieties, an equivalent artificial sugar solution of 64 °Brix was produced and adjusted to a pH of 3.2 with a citric acid buffer. The sucrose, glucose and fructose composition was chosen according to ref. ^45^ and is shown in Supplementary Table 2. To ensure the dissolution of the sugars, the mixture was stirred for 90 min at 70 °C.

### ECP gels

ECP was added to the artificial sugar solution at 5 wt%, 10 wt%, 20 wt% and 25 wt%. The samples were stirred for 10 min by hand and left to rest for 10 min. The pH was measured using a porotrode (Metrohm) and adjusted to 3.2 using 1–12 M HCl. The samples were then heated to 80 °C at 2.5 °C min^−1^ and left at 80 °C for 16 min before being analysed in the rheometer or allowed to cool at room temperature to 31 ± 0.5 °C for the kneading experiments.

### Whole-fruit chocolate formulations

The ECP gels were mixed with the CM in a kneader containing two intermeshing blade rotors positioned in a double cylindrical mixing chamber (IKA Kneader MKD 0.6 H60; IKA Werke). The dimensions of the blades and the chamber are shown in Supplementary Fig. 2 and Supplementary Table 6. During kneading, the torque was measured by a brushless torque sensor and analysed with the LabworldSoft 4.01 software (labworld-online). First, molten CM at 50 °C was poured into the pretempered kneader operated at 38 rpm and equilibrated for 10 min. The CM was cooled to 26 °C over 90 min to induce crystallization. Subsequently, the CM temperature was raised to 31 °C to induce the formation of *β*_V_ polymorphic CB crystals. After 15 min at 31 °C, the pretempered gels were added to the CM at 1.6 g min^−1^. Kneading was performed until the torque remained constant. CM:gel ratios were varied to 95:5, 90:10, 80:20 and 70:30. The total kneading mass was 312 g for all ratios. The resulting CM–ECP gel samples were stored at 50 °C to remelt the formed crystals.

### Photography

CM–ECP gel systems were taken out of the IKA kneader and heated to 50 °C for 24 h in plastic cups placed in an oven. A spoon of the CM–ECP gel system was placed on a petri dish. The petri dish was placed in a photo box with uniform lighting and a picture was taken using an iPhone XS camera running on iOS 15. The images were exported to Adobe Illustrator 2021 and processed.

### Rheological analysis of the ECP gels

An oscillatory shear rheometer (MCR 302; Anton Paar) was used. Gel samples at 80 °C were placed on a sandblasted plate–plate *PP*25 geometry of 25-mm plate diameter set-up preheated to 80 °C. The upper plate was lowered to a 1 mm gap and the sample trimmed. Equilibration at 80 °C was performed for 10 min before cooling the sample to 31 °C at 1 °C min^−1^ with an oscillation frequency *ω* of 1 rad s^−1^ and a deformation *γ* of 0.1%. The sample was left at 31 °C for 30 min before conducting a frequency sweep from 1rad s^−1^ to 100 rad s^−1^ (*γ* = 0.1%) followed by an amplitude sweep from 0.1% to 1,000% (*ω* = 1 rad s^−1^). The amplitude sweep was followed by a 30 min recovery step at 31 °C (*ω* = 1 rad s^−1^, *γ* = 0.1%). Measurements were performed in triplicate (*n* = 3). The software RheoCompass 1.31.70 by Anton Paar was used to gather the data, and data were exported to Excel 2021 for calculating the mean and s.d. Data were plotted in OriginPro 2021 and illustrated using Adobe Illustrator 2021.

### Rheological analysis of chocolate

A shear rheometer (MCR 302; Anton Paar) equipped with a CC27 Couette system was used. The chocolate was stored at 50 °C for 24 h and then transferred to the preheated CC27 cup. Temperature was equilibrated for 180 s (±0.2 °C) before a preshear of 5 s^−1^ was applied for 180 s. Afterwards, a logarithmic shear rate ramp from 1 s^−1^ to 500 s^−1^ within 27 steps was performed upwards (1–500 s^−1^) and downwards (500–1 s^−1^) with an intermediate step of 180 s at 500 s^−1^. Measurements were performed in triplicate (*n* = 3). The software RheoCompass 1.31.70 by Anton Paar was used to gather the data, and data were exported to Excel 2021 for calculating the mean and s.d. Data were plotted in OriginPro 2021 and illustrated using Adobe Illustrator 2021.

### Water activity

*a*_w_ was measured with the LabMaster AW Neo (Novasina). The gels (80 °C) were left to cool down at room temperature before they were put into a cylindrical sample holder for measurement. Measurements were performed in triplicate (*n* = 3) at 25 °C with a set stability time of 4 min. The verification tolerance of the device was ±0.005. Data were collected by reading out the value indicated on the screen of the device by eye. Data evaluation was done in Excel 2021 by calculating the mean and s.d. of the triplicates. Data were plotted in OriginPro 2021 and illustrated using Adobe Illustrator 2021.

### Moisture content

Moisture content of the ECP was measured with a HR73 Halogen Moisture Analyzer (Mettler Toledo). The temperature was set to 120 °C. Program 3 was selected, which dries the sample until the weight change over time reaches a plateau. Data were collected by reading out the value indicated on the screen of the device by eye. Data evaluation was done in Excel 2021 by calculating the mean and s.d. of the triplicates. Data were plotted in OriginPro 2021 and illustrated using Adobe Illustrator 2021.

### Particle size distribution

The LS13 320 laser diffraction particle size analyser (Beckman Coulter) with a universal liquid module was applied. A continuous phase Hydriol SOD.24 (Hydrior) was used. The diffraction pattern was interpreted using the Fraunhofer model. Polarization intensity differential scattering data was included. At least three replicates were performed and averaged. The ECP was mixed with 10 wt% MCT oil (Witarix MCT 60/40; Oleochemicals) and sonicated with a sonotrode of 5 mm (UP 200H; Hielscher Ultrasound Technology) at 60 Hz for 2 min. Measurements were performed in triplicate (*n* = 3). The obscuration was around 6% and pump speed was set to 70%.

### Whole-fruit chocolate formulations for 2-AFC sensory evaluation

CPJC (50 °Brix and 72 °Brix) from Assin Akrofuom (Ghana) was provided by Koa Switzerland. The CPJCs were blended to reach 64 °Brix and heated to 80 °C in a pan. The fine-milled ECP (Martin Bauer Group) was mixed at 25 wt% into the CPJC using a Hobart planetary bowl mixer (Hobart A200; Hobart). The resulting CPJC gel was homogenized into liquid CM (Max Felchlin) using a bottom blade bowl blender (Robot Coupe R8; Robot Coupe) at CM:gel weight ratios of 80:20 to produce the whole-fruit chocolate. The chocolate was tempered to 31–34 °C and seeded with CB (Max Felchlin AG) held at 34 °C for 2–4 h before being moulded into 5 g discs using a polycarbonate mould with length × breadth × height of 275 mm × 135 mm × 24 mm, including 3 × 7 = 21 discs of 35 mm diameter per mould (Max Riner).

### 2-AFC sensory evaluation

Eleven trained panellists (11 women aged 38–56) participated in the 2-AFC study. Panellists were unknowingly confronted multiple times with the same sample choices. All tests were conducted at the sensory laboratory of the University of Applied Science Bern (BFH-HAFL). The test was divided into four blocks, each one containing four pairs of samples per panellist in a completely randomized order (according to ISO 5495:2005). Before the test sessions, all samples were placed on white plates at room temperature (25 °C) in a balanced design. All samples were evaluated in sensory booths at room temperature and normal light conditions. The panellists were allowed to apply a red light, in case visual difference could mask judgement, although weight and shape was standardized to 5 g discs. Subjects were instructed to place the smooth side of the sample in the mouth, melt it for approximately 3 s and slowly chew it. There were no restrictions in swallowing or complete chewing of the sample, as long as consistency in individual tasting protocol was guaranteed. All panellists received the samples as pairs in a balanced test design and were asked to indicate the sweetest sample of each pair after tasting both samples. Still water (25 °C) and unsalted crackers were provided to clean the palate between samples. Breaks of 10 min were taken between each replication block. Data were collected with Eye Question (licensed to BFH-HAFL) at a test power of *β* = 0.10 and a statistical probability of choosing either control or reference of *µ* = 0.5. Data were analysed using EyeOpenR and all data from the directional paired comparison test were evaluated two tailed. Differences were evaluated as significant at an *α* level of 0.05. Data were plotted in OriginPro 2021 and illustrated using Adobe Illustrator 2021.

### LCA

We conducted an LCA from cradle to factory using OpenLCA and the ReCiPe 2016 Midpoint (H) impact assessment method^46^. We compared the whole-fruit chocolate against two benchmark chocolates because the environmental impact of chocolate is largely dominated by the amount of CM in the product; thus, the choice of product formulation will drastically affect results. The first benchmark, denoted Euro avg, is representative of an average dark chocolate sold on the European market^19^. However, it is also imperative to compare the whole-fruit chocolate against a product with a similar formulation. Accordingly, we created the substitute benchmark to have the same CM and equal sugar content as F2; moreover, we also matched the cocoa powder to the ECP amount as shown in Supplementary Table 4. We then confirmed that a chocolate with a similar formulation existed on the market so that F2 is compared against chocolates currently sold and thus accepted by consumers. The whole-fruit chocolate formulation F1 was excluded from the LCA analysis as it was assumed to be a non-competitive product based on consumer preference. Our system boundaries, as shown in Supplementary Fig. 3, included farming, transportation, processing and packaging. Processing steps were determined based on input from the producers. We did not include end of life as we assume this to be the same for both products; relatedly, we assumed the cocoa beans for all four chocolates were cultivated on a Ghanaian farm with similar farming practices. The data sources are in Supplementary Table 7 and inputs in terms of energy, water, heating and materials are presented in Supplementary Table 8. This is a new product produced in a lower-middle-income country; consequently, limited measurement data exist and data were calculated based on primary information from the producers or on equipment specifications. We included two scenarios for the whole-fruit chocolate; the current or laboratory scale and the industrial scale (that is, at scale) scenario. The former is a mix of both laboratory and industrial scale because two processes are already completed at scale (that is, fine milling and juice concentrating). The pulp and ECP are normally waste products that are left on the field after cocoa bean harvesting. Thus, they were treated as zero burden in this model, and the only impacts ascribed to them were those due to transportation from the farms to the factory in Ghana. The endocarp and pulp are removed by hand from the cocoa pods and are then processed in a factory. For the ECP, we calculated the amount of energy needed to dry the product based on equation (1). The energy for milling the endocarp was measured in a previous study^47^. Natural gas was used for drying and a mix of grid, solar and diesel was used for premilling. At scale, we assumed the diesel would be replaced by solar energy. Efficiencies for convective dryers are on the lower side compared with other processes, and based on the literature, airspeed values for dryers similar to ours range from 0.5 m s^−1^ to 2 m s^−1^. For our set-up, we assumed an airspeed of 1.0 m s^−1^ and calculated an efficiency value of 10.6% based on a regression determined from a previous study’s data^48^. The endocarp drying process is still in the experimental phase and it is unclear what a set-up at scale would look like. However, we do know that the efficiency would probably increase and so we assume an efficiency of 45%, which is a value on the higher end for convective dryers. Moisture content of the endocarp pre- and postdrying was measured in the laboratory. The energy was then adjusted per kilogram of dried endocarp. For the juice concentrate, pressing was calculated based on equipment specifications, and we assumed an efficiency of 21%, which is an average value for hydraulic presses^49^, and pasteurization and cooling were calculated based on equation (2); an efficiency of 80% was used. At scale, pasteurization and thus energy requirements are higher to avoid the need for cooled transport. Solar energy is used for pressing whereas a mix of solar and grid energy is used for pasteurization and cooling. The energy required for concentrating the juice was adapted from the ecoinvent process for milk evaporation. For the gel, the energy for mixing was provided by the producer for the laboratory-scale scenario and at scale we assume a mixing energy based on ref. ^50^ for a 1,000 l set-up with a radial flow (equation (3)). We calculated the energy for heating based on equation (4)^50^. For the laboratory-scale scenario, we used the area dimensions of our current set-up and for the at-scale scenario we used the values given in ref. ^50^ for a 1,000 l set-up. The specific heat capacity (*c*_p2_) of the gel was calculated based on ratio mixes (equation (5)). For transport, we used ref. ^51^ to determine distances and emission standard types. All products were trucked across land unless being shipped from Ghana to Europe. For cooled transport, we assumed refrigeration specific to the transportation type.

1 $${E}_{1}=\frac{{m}_{\rm{i}} {c}_{\rm{p}} \Delta T+{m}_{\rm{l}} \Delta {H}_{\rm{vap}}}{\phi }$$

2 $${E}_{2}=\frac{{m}_{\rm{i}} {c}_{\rm{p}} \Delta T}{\phi }$$

3 $${E}_{3}=\frac{{N}_{\rm{p}} {\rho }_{\rm{i}} {N}^{3} {d}^{5} t}{\phi }$$

4 $${E}_{4}=\frac{\Delta T({m}_{\rm{i}} {c}_{\rm{p}2}+A \frac{{k}_{\rm{a}}}{s} t)}{\phi }$$

5 $${c}_{\rm{p}2}=\mathop{\sum }\limits_{i=1}^{j}{c}_{{\rm{p}}i} {w}_{i}$$

where *E* = energy (J), *ρ* = density (kg m^−3^), *m*_i_ = product mass (kg), *c*_p2_ = specific heat capacity (J kg^*−*1^ K^−^^1^) (of the gel), *w*_i_ = mass fraction of species *i* with respect to the total mass of a mixture with *j* species, *m*_l_ = evaporated water mass (kg), ∆*T* = temperature difference between ambient and drying or heating temperature (K), ∆*H*_vap_ = water latent heat of evaporation (J kg^*−*1^), *t* = time (s), *A* = surface area (*m*^2^), *k*_a_ = thermal conductivity of insulation (W m^−^^1^ K^−^^1^), *s* = insulation thickness (m), *N*_p_ = dimensionless stirrer constant, *N* = rotational velocity (s^−^^1^), *d* = impeller diameter (m) and *Φ* = efficiency. For our study, we used physical allocation, with allocation factors presented in Supplementary Table 9. Economic allocation was not used because allocation factors can vary considerably depending on market prices. The prices per ton of our coproducts can vary greatly across and within years; for instance, CB pricing can differ substantially based on demand from the cosmetic industry^52^. As all coproducts (CM, CB and cocoa powder) in our system are of a high quality, mass-based allocation was used. Cocoa shells can be used for energy^53^; however, in our system, shells are not sold for this purpose. Regardless, the economic value is minimal and would not affect results^2^. Data were collected using OpenLCA and analysed using ReCiPe 2016 Midpoint (H). Data were plotted in OriginPro 2021 and illustrated using Adobe Illustrator 2021.

### Ethics declaration

All participants provided written informed consent. This study has been submitted to the ethical advisory board of the University of Applied Sciences Bern (EAB2024_005). The advisory board determined that the study did not raise ethical concerns as no clinical data were collected.

### Reporting summary

Further information on research design is available in the Nature Portfolio Reporting Summary linked to this article.

### Supplementary information

Supplementary Information Supplementary Tables 1–9, Figs. 1–3 and Method 1.

Reporting Summary

### Source data

Source Data Fig. 2 **a**, Source data of time (abs., rel.), temperature, mean *G*′ and *G*″, and s.d. *G*′ and *G*″ for sweetening gels with 5, 10, 20 and 25 wt% ECP. **b**, Source data of amplitude of deformation, time, mean *G*′ and *G*″, and s.d. *G*′ and *G*″ for sweetening gels with 5, 10, 20 and 25 wt% ECP. **c**, Source data of mean *G*′ and *G*″ and s.d. *G*′ and *G*″ for sweetening gels with 5, 10, 20 and 25 wt% ECP. **d**, Source data of mean and s.d. of *a*_w_ for sweetening gels with 5, 10, 20 and 25 wt% ECP.

Source Data Fig. 3 Original, uncropped images of the simplified chocolate formulations displayed in Fig. 3. Grouped according to ECP concentration in gel.

Source Data Fig. 4 Source data of mean and s.d. of viscosity as a function of shear rate for simplified chocolate formulations.

Source Data Fig. 5 Source data for Fig. 5.

Source Data Fig. 6 Source data, *P* values, information, raw data, product comparisons, assessors and design control for Fig. 6a,b.

Source Data Fig. 7 Source data for Fig. 7a,b.

## Data Availability

All source data shown in Figs. 2–7 are provided with this paper.
