# Supplementary material for: Valorization of cocoa pod side streams improves nutritional and sustainability aspects of chocolate
Source: Nat Food. 2024 May 21;5(5):423–32. doi: 10.1038/s43016-024-00967-2 (PMC11132982; doi:10.1038/s43016-024-00967-2)
Supplement: Supplementary file 1 — Supplementary Tables 1–9, Figs. 1–3 and Method 1. [file 43016_2024_967_MOESM1_ESM.pdf]

nature food

Article

https://doi.org/10.1038/s43016-024-00967-2

Valorization of cocoa pod side streams improves nutritional and sustainability aspects of chocolate

In the format provided by the authors and unedited

Supplementary Table 1: **Composition of the cocoa pod endocarp.** The pectin extraction in this study was performed at 90°C and pH=2.5 for 2.5 h by Herbstreith and Fox GmbH & Co. KG. Germany.

Fraction [g/kg dry matter]	Fresh EC[1]	Fresh EC (this study)	ECP (this study)
Moisture content	871.0±2.1	864	38.3±1.6
Crude protein	91.0±3.0	86	
Total ash	59.0±4.0	67	
Hemicellulose	70.0±1.0	-	37.4±18.9
Cellulose	208.0±3.0	-	105.6±26.0
Lignin	132.0±1.0	-	226.8±46.7
Pectin	105.0±2.0	-	124-142
Based on Pectin fraction			
Galacturonic acid	-	69.3	61.8-64.9
Degree of esterification	-	60.8	51.1-52.7
Degree of methylation	-		5.0-5.5
Degree of acetylation	-	16.9	0-2.83
Molecular weight (g/mol)	-		16774-24431

Supplementary Table 2: **Artificial sugar solution composition.** The artificial sugar solution was used for the study of the sweetening gels' material properties and colloidal stability of simplified chocolate formulations.

Compound	Concentration [wt%]	Producer
deionized Water	33.78	ETH Zürich, Zürich, Switzerland
citric acid monohydrate	1.81	Merck KGaA, Darmstadt, Germany
sodium citrate dihydrate	0.41	Merck KGaA, Darmstadt, Germany
saccharose	25.0	Schweizer Zucker AG, Frauenfeld, CH
glucose	17.2	Roquette Frères, Lestrem, France
fructose	21.8	Migros-GB, Zürich, Switzerland

Supplementary Table 3: **Detailed composition of the studied chocolates in the 2-AFC sensory test.** Calculated composition in wt% of samples compared in the sensory 2-AFC test. F1 and F2 were compared with pure CM and CM containing half (CM + 0.5PS), equal (CM + 1PS) or one and a half (CM + 1.5 PS) the equivalent of the F1/F2 sugar content as PS. The exact composition of the CM without added sugar is listed in Table 5.

Material	Origin	F1	F2	CM + 0.5 PS	CM + 1 PS	CM + 1.5 PS
Cocoa mass	Ghana	80	68	95.2	90.4	85.6
Cocoa butter	Ghana	0	12	0	0	0
Powdered sugar	CH	0	0	4.8	9.6	14.4
CPJC gel	Ghana	20	20	0	0	0
Total fat	-	44.0	49.4	52.36	49.74	47.08
Total sugars	-	9.6	9.6	4.8	9.6	14.4
Total fibers	-	16.86	14.76	16.66	15.82	14.98

Supplementary Table 4: **Fiber and SFA content of chocolates compared in the life-cycle analysis.** The fiber and saturated fatty acid (SFA) content in wt% of cocoa mass, cocoa butter, powdered sugar, cocoa powder, and CPJC gel and their used wt% in the chocolate formulations F2, Euro avg, and Substitute. The exact composition of the CM without added sugar is listed in Table 5. Total fiber, SFA, sugar, and ECP content for F2, Euro avg, and Substitue are given.

Material	fiber	SFA	F2	Euro avg	Substitute
Cocoa mass	17.5	33.5	68	42	68
Cocoa butter	0	61	12	28	17.4
Powdered sugar	0	0	0	14	9.6
Cocoa powder	28	13	0	16	5
CPJC gel	14.4	0	20	0	0
Total fibers	-	-	14.8	11.8	13.3
Total SFAs	-	-	22.8	33.2	34.0
Total sugar	-	-	9.6	14	9.6
Total ECP	-	-	5	0	0

Supplementary Table 5: **Cocoa mass composition.** The composition is specified in g/g for fats (i.e. triacylglycerides), carbohydrates, dietary fibers, and proteins. The total fat content is broken down into saturated-, monounsaturated-, and polyunsaturated fatty acids. Carbohydrate composition is broken down into sugars (i.e. mono- and disaccharides) and saccharides with higher degree of polymerization.

Compound	Concentration [g/100g]
Fats	55
of which saturated fatty acids	33.5
of which monounsaturated fatty acids	20.4
of which polyunsaturated fatty acids	1.1
Carbohydrates	8.8
of which sugars	0.3
Fibers	17.5
Proteins	11.1

Supplementary Method 1: **Fiber analysis**

Neutral detergent fiber (NDF): The neutral detergent solution (NDF) solution (5L) was prepared with 50 mM $C_10H_14N_2Na_2O_8$ + $2H_2O$ (titriplex III di-sodium salt), 100 mM $CH_3(CH_2)11SO_4Na$ (sodium dodecylsulfate), 18 mM $Na_2[B_4O_5(OH)_4]$ + $8H_2O$ (di-sodium tetraborate decahydrate), 32mM Na_2HPO_4 (di-sodium hydrogen phosphate), and 73 mM $C_6H_14O_4$ (triethylene glycol) taken to 5 L of deionized water. The final solution had a pH of 7. The NDF analysis was conducted using a fiberbag system (Fibretherm, Gerhardt Analytical Systems, Germany). A sample of 0.5 g is weighed and transferred into the fibrebag. The process consists of a boiling phase of the detergent solution, followed by two phases of boiling water used to rinse the solution in order to fully remove every neutral soluble compounds from the samples and takes approximately 2 h. Due to the detergent foaming nature of the solution, excessive foam presented a problem in the first trials, until it was standardized to add 4 ml of antifoam solution (Sigma-Aldrich, Switzerland) before beginning the process. After the digestion and rinsing phases, each bag was rinsed with deionized water in order to remove the spreader allocated inside of the bag (accessory that aids the weighing and digestion stages) while keeping all the neutral insoluble remains in the bag. Then, the bags

were placed in glass crucibles and dried in the oven at either 105°C overnight or 130°C for 2 h. After cooling them down in a desiccator, the samples were weighed and allocated in the firebag system to continue the process with ADF. *Acid neutral detergent (ADF):* The acid neutral detergent (ADF) solution (5 L) was prepared with 520 mM of H_2SO_4 96% concentrated (sulfuric acid) and 55 mM of C_19H_42BrN (Cetyltrimethylammonium bromide) taken to 5 L of deionized water. The final solution had a pH of 1. The conditions and parameters for ADF methodology are the same as the ones described for NDF. The only difference is that the acid detergent solution mentioned before was used instead of a neutral detergent solution. After this, the bags were dried in the oven at either 105°C overnight or 130°C for 2 h, cooled and weighed. Then the bags, containing the acid insoluble fraction of the sample, were allocated in a specific carousel to conduct acid detergent lignin. *Acid detergent lignin (ADL):* This step consisted of soaking the samples in 250 ml of 72% sulfuric acid (Merck, purity 96-98%) for 3 h under a chemical fume hood. Afterwards, each sample was rinsed with deionized water until all the acid was removed. To ensure this, they were washed until the draining water reached a neutral pH of 6-8, measured using the digital pH meter. Then the bags were dried in the oven at either 105°C overnight or 130°C for 2 h. Then they were cooled in a desiccator and the weight was recorded. After this, the bags were allocated in the glass crucibles for the final step, which consisted of determining the ash content. A muffle furnace was used (LE 14/11 Nabertherm, Bremen, Germany) at 500°C overnight. After obtaining the values for NDF, ADF, and ADL, the lignocellulosic fractions were obtained using the following equations (Hindrichsen et al., 2006; Van Soest et al., 1991). The cellulose, hemicellulose and lignin %DM content were calculated as:

$$Cellulose = ADF - ADL \tag{1}$$

$$Hemicellulose = NDF - ADF \tag{2}$$

$$Lignin = ADL \tag{3}$$

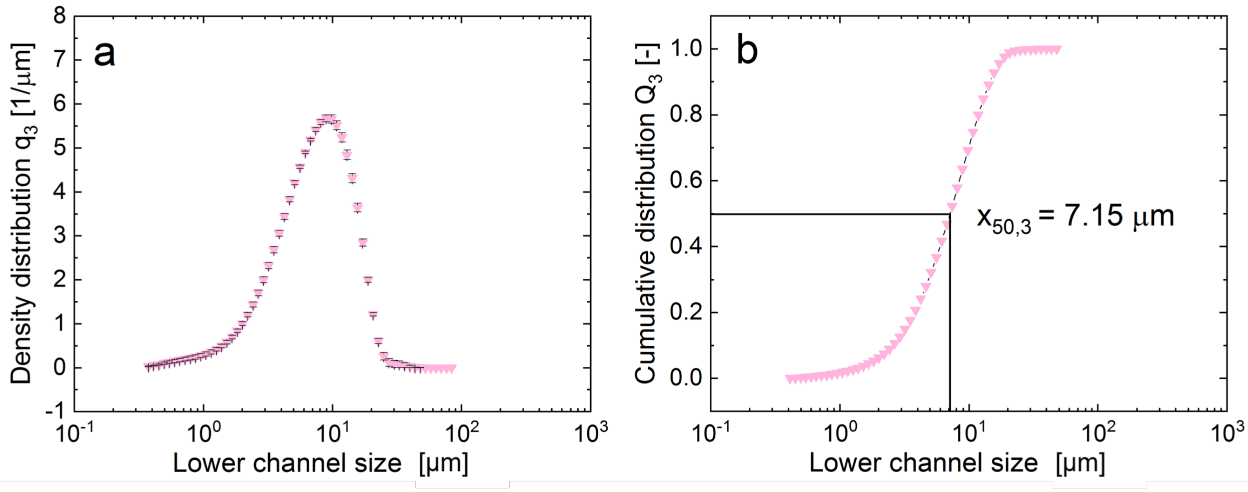

Supplementary Figure 1: **ECP particle size and size distribution.** The (a) density as well as the (b) cumulative size distribution q_3/Q_3 of the cocoa pod endocarp powder (ECP) used in this study.

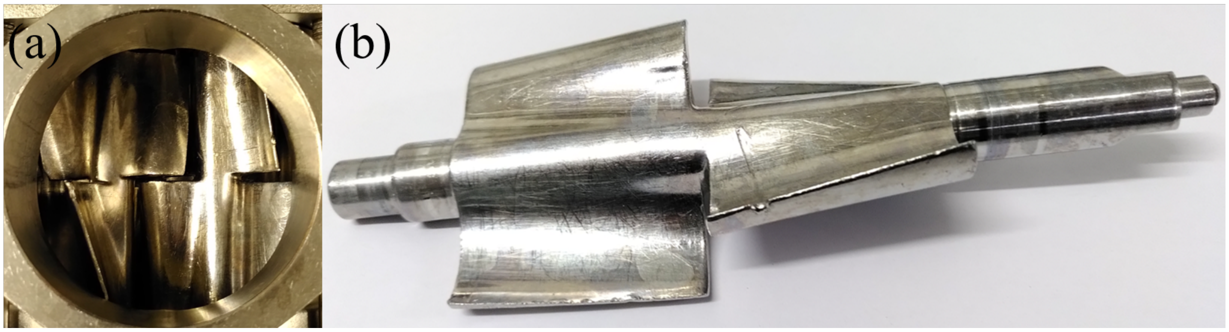

Supplementary Figure 2: **IKA kneader images.** View from above displaying the two intermeshing shafts in the chamber (a) and the isolated shaft (b).

Supplementary Table 6: **IKA kneader dimensions.** The dimensions of the single shaft, the two mounted shafts together and the dimensions of the chamber are given.

Diameter of a shaft [cm]	5.1
Width of two mounted shafts [cm]	7.9
Length of a shaft [cm]	8.9
Width of the mixing chamber [cm]	9.1
Length of the mixing chamber [cm]	10.4

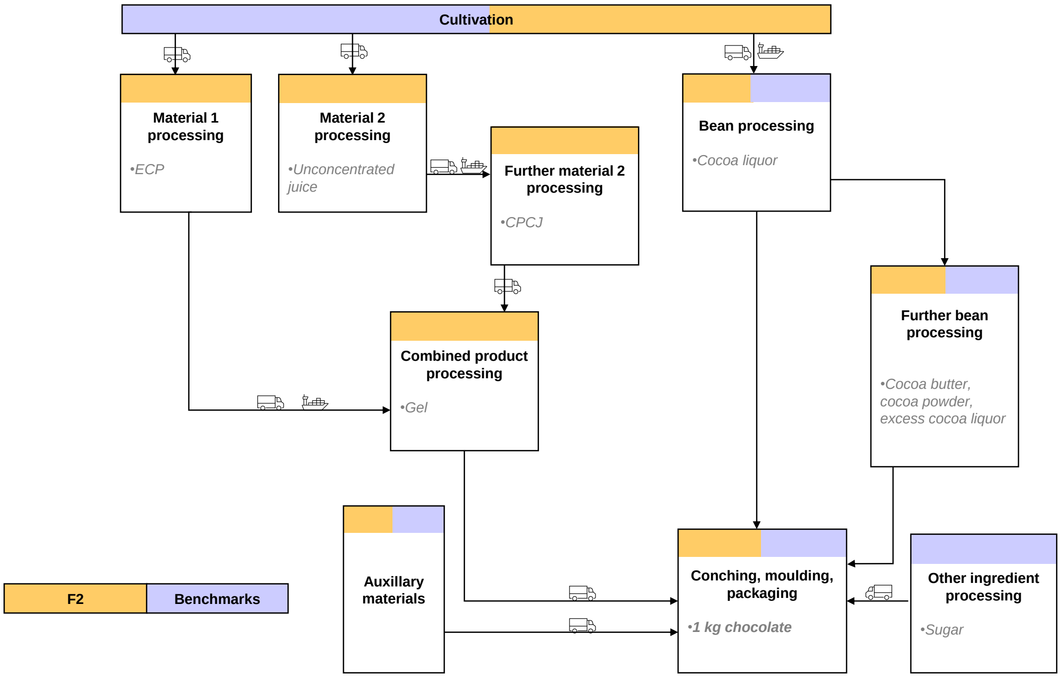

Supplementary Figure 3: **Systems boundaries for whole fruit and benchmark chocolates.** Cocoa liquor is equivalent to cocoa mass. The truck or ship icon between the steps indicate transportation methods.

Supplementary Table 7: **Data types and sources used in the LCA.** Ecoinvent refers to a LCA database. Primary refers to data collected in this study.

Phase	Source
Cocoa beans (Ghana)	ecoinvent
Endocarp processing	Drying: primary,
(values per 1 kg	Pre-milling lab scale: Grob et al.[2]
of endocarp powder)	Pre-milling at scale: Piccinno et al.[3]
	Fine-milling: primary
1 kg CPJ	Primary
1 kg CPJC	Pressing at scale: Zimmer et al.[4] Other steps: primary
Gel	Primary
CM (bean processing)	Recanati et al.[5]
CP and CB (bean processing)	Recanati et al.[5]
Chocolate production	Recanati et al.[5]
Sugar	ecoinvent
Transport	ecoinvent

Supplementary Table 8: **Life cycle inventory input data.** CPJ = Cocoa pulp juice, CPCJ = Cocoa pulp juice concentrate, CM = Cocoa mass, CP = Cocoa powder, CB = Cocoa butter, CS = Cocoa shells.

Process	Position	F2 lab scale	Benchmarks	F2 at scale
**ECP**			-	
	electricity	1.5005 kWh	-	0.176 kWh
	natural gas	111.18 MJ	-	26.2 MJ
outputs		*ECP 1 kg*	-	*ECP 1 kg*
**CPJ**				
	electricity	0.379 kWh	-	0.2 kWh
output		*CPJ 1 kg*	-	*CPJ 1 kg*
**CPJC**				
	electricity	0.011 kWh	-	0.011 kWh
	heat	0.707 MJ	-	0.707 MJ
	water	0.27 kg	-	0.27 kg
output		*CPJC 1 kg*	-	*CPJC 1 kg*
**Gel**				
	electricity	0.079 kWh	-	0.044 kWh
output		*Gel 1 kg*	-	*Gel 1 kg*
**Bean Processing**				
	electricity	0.505 kWh	0.505 kWh	0.505 kWh
	water	0.7 kg	0.7 kg	0.7 kg
	lubricating oil	24.2 mg	24.2 mg	24.2 mg
	natural gas	0.502 MJ	0.502 MJ	0.502 MJ
output		*CM 501 g*	*CM 501 g*	*CM 501 g*
		CS 76 g	*CS 76 g*	*CS 76 g*
**Further bean processing**				
	electricity	0.364 kWh	0.364 kWh	0.364 kWh
	water	0.9 kg	0.9 kg	0.9 kg
	lubricating oil	17.6 mg	17.6 mg	17.6 mg
	natural gas	0.323 MJ	0.323 MJ	0.323 MJ
outputs		*CP 160 g*	*CP 160 g*	*CP 160 g*
		CB 202 g	*CB 202 g*	*CB 202 g*
		CM 98 g	*CM 98 g*	*CM 98 g*
		CS 55 g	*CS 55 g*	*CS 55 g*
**Chocolate Production**				
	electricity	0.97 kWh	0.97 kWh	0.97 kWh
	water	1.7 kg	1.7 kg	1.7 kg
	lubricating oil	46.2 mg	46.2 mg	46.2 mg
	natural gas	0.197 MJ	0.197 MJ	0.197 MJ
output		*chocolate 1 kg*	*chocolate 1 kg*	*chocolate 1 kg*

Supplementary Table 9: **Mass allocation factors used in the LCA.** CM = cocoa mass, CB = cocoa butter, CP = cocoa powder.

Co-product	Allocation factor
CM	0.21
CB	0.44
CP	0.35

References

(1) Sobamiwa, O.; Longe, O. *Animal Feed Science and Technology* **1994**, *47*, 237–244.

(2) Grob, L.; Ott, E.; Schnell, S.; Windhab, E. J. *Journal of Food Engineering* **2021**, *305*, 110591.

(3) Piccinno, F.; Hischier, R.; Seeger, S.; Som, C. *Journal of Cleaner Production* **2016**, *135*, 1085–1097.

(4) Zimmer, E.; others *Fruit Processing* **2017**, *27*, 12–16.

(5) Recanati, F.; Marveggio, D.; Dotelli, G. *Science of The Total Environment* **2018**, *613*, 1013–1023.
