# Supplementary material for: Valorization of cocoa pod side streams improves nutritional and sustainability aspects of chocolate
Source: Nat Food. 2024 May 21;5(5):423–32. doi: 10.1038/s43016-024-00967-2 (PMC11132982; doi:10.1038/s43016-024-00967-2)
Supplement: Supplementary file 2 — Reporting Summary [file 43016_2024_967_MOESM2_ESM.pdf]

nature portfolio

Corresponding author(s): Kim Mishra

Last updated by author(s): Mar 10, 2024

Reporting Summary

Nature Portfolio wishes to improve the reproducibility of the work that we publish. This form provides structure for consistency and transparency in reporting. For further information on Nature Portfolio policies, see our Editorial Policies and the Editorial Policy Checklist.

Statistics

For all statistical analyses, confirm that the following items are present in the figure legend, table legend, main text, or Methods section.

n/a	Confirmed	
☐	☒	The exact sample size (*n*) for each experimental group/condition, given as a discrete number and unit of measurement
☐	☒	A statement on whether measurements were taken from distinct samples or whether the same sample was measured repeatedly
☐	☒	The statistical test(s) used AND whether they are one- or two-sided<br>*Only common tests should be described solely by name; describe more complex techniques in the Methods section.*
☒	☐	A description of all covariates tested
☒	☐	A description of any assumptions or corrections, such as tests of normality and adjustment for multiple comparisons
☐	☒	A full description of the statistical parameters including central tendency (e.g. means) or other basic estimates (e.g. regression coefficient) AND variation (e.g. standard deviation) or associated estimates of uncertainty (e.g. confidence intervals)
☐	☒	For null hypothesis testing, the test statistic (e.g. *F*, *t*, *r*) with confidence intervals, effect sizes, degrees of freedom and *P* value noted<br>*Give P values as exact values whenever suitable.*
☒	☐	For Bayesian analysis, information on the choice of priors and Markov chain Monte Carlo settings
☒	☐	For hierarchical and complex designs, identification of the appropriate level for tests and full reporting of outcomes
☒	☐	Estimates of effect sizes (e.g. Cohen's *d*, Pearson's *r*), indicating how they were calculated

Our web collection on statistics for biologists contains articles on many of the points above.

Software and code

Policy information about availability of computer code

Data collection	Figure 2(a-c): RheoCompass 1.31.70 by Anton Paar, Figure 2(d): Read out by eye from screen of LabMaster neo, Figure 3: Kneading of simplified chocolate formulations by LabworldSoft 4.01 software, Images by Iphone Xs camera run on iOS 15 by Apple, Figure 4(a-d): RheoCompass 1.31.70 by Anton Paar, Figure 5: No primary data, Figure 6: Eye Question 5.1, Figure 7: OpenLCA, Supp. Table 1: Moisture content data was collected by reading out the HR73 screen by eye, fiber data collected by reading out display of gravimetric scale by eye, Supp. Fig. 1:Particle size primary data was given in Excel 2021.
Data analysis	Fig.1: Completely done in Adobe Illustrator 2021, Fig2(a-d), Fig4, Fig5:Analysis in Excel 2021, Plotting in OriginPro 2021, Illustation in Adobe Illustrator 2021 (AI 2021), Fig.3: Kneading of simplified chocolate formulations in Excel, AI 2021 for images Figure 6: EyeOpenR, Excel 2021, OriginPro 2021, AI 2021 Figure 7: Recipe 2016 Midpoint (H), Excel 2021, OriginPro 2021, AI 2021, Supp. Table 1: Excel 2021, OriginPro 2021, Supp. Fig. 1: Excel 2021, OriginPro 2021

For manuscripts utilizing custom algorithms or software that are central to the research but not yet described in published literature, software must be made available to editors and reviewers. We strongly encourage code deposition in a community repository (e.g. GitHub). See the Nature Portfolio guidelines for submitting code & software for further information.

Data

Policy information about availability of data

All manuscripts must include a data availability statement. This statement should provide the following information, where applicable:

- Accession codes, unique identifiers, or web links for publicly available datasets
- A description of any restrictions on data availability
- For clinical datasets or third party data, please ensure that the statement adheres to our policy

All source data of Fig (2-7) is supplied and listed in the Inventory of Supporting Information.

Human research participants

Policy information about studies involving human research participants and Sex and Gender in Research.

Reporting on sex and gender	Sex of 11 trained panelists is female
Population characteristics	Biological age is between 38-56
Recruitment	Recruitment was done internally at the University of applied science Bern
Ethics oversight	Exemption from ethical approval was given by the ethics commission of the university of applied science Bern (EAB2024_005). Participants gave informed consent via the statement “I am aware that my responses are confidential, and I agree to participate in this survey” where an affirmative reply was required to enter the survey. They were able to withdraw from the survey at any time without giving a reason. The products tested were safe for consumption.

Note that full information on the approval of the study protocol must also be provided in the manuscript.

Field-specific reporting

Please select the one below that is the best fit for your research. If you are not sure, read the appropriate sections before making your selection.

☒ Life sciences ☐ Behavioural & social sciences ☐ Ecological, evolutionary & environmental sciences

For a reference copy of the document with all sections, see nature.com/documents/nr-reporting-summary-flat.pdf

Life sciences study design

All studies must disclose on these points even when the disclosure is negative.

Sample size	Sample size for rheological analysis was set to 3 as commonly done with rheometrical measurements. Number of observations for the sensory study was calculated for a two-sided binomial test based on ISO 5495:2005, power of discrimination pd = 0.5, alpha = 0.05, and beta = 0.1 to be 42. We used 11x4 = 44 observations. Power of beta = 0.1 and statistical power = 1-b = 0.9. Based on Chapter 6 of ISO 5495 2005 (E), all assessors/panelists are considered qualified rather than naive, as they have undergone repeated training with the method (2-AFC) and similar products (e.g., chocolate) (ISO 8656-1/2). Based on internal experience, a Pd (proportion of the population of assessors who can differentiate between the two samples) between 40-50% was therefore defined. Together with the alpha value (5%) and the beta risk (10%), according to ISO standard based on [1], a theoretical #number of observations value of ~40 is obtained. In summary, based on internal experience, regular training of the panel with this method and similar products, a Pd as defined above was deemed sufficient without the need for prior blinded re-evaluation of the sample size on a small scale. [1]: SCHLICH, P. Risk Tables for Discrimination Tests. Food Quality and Preference, 4, 1993, pp. 141-151
Data exclusions	No data was excluded
Replication	Experimental repeatability for data presented in Figure 2 and 4 was done by triplicate measurements. All attempts at replication were successful. Images shown in Figure 3 display only one repetition. All attempts at replication were successful. Repeatability for the data in Figure 6 was ensured by performing 4 replicates. All attempts at replication were successful.
Randomization	Chocolate samples given to panelists were randomized according to ISO 5495:2005
Blinding	One-sided blinding was done (participants did not know which samples they got)

Reporting for specific materials, systems and methods

We require information from authors about some types of materials, experimental systems and methods used in many studies. Here, indicate whether each material, system or method listed is relevant to your study. If you are not sure if a list item applies to your research, read the appropriate section before selecting a response.

Materials & experimental systems

n/a	Involved in the study	
☒	☐	Antibodies
☒	☐	Eukaryotic cell lines
☒	☐	Palaeontology and archaeology
☒	☐	Animals and other organisms
☒	☐	Clinical data
☒	☐	Dual use research of concern

Methods

n/a	Involved in the study	
☒	☐	ChIP-seq
☒	☐	Flow cytometry
☒	☐	MRI-based neuroimaging
