# Supplementary figures and images for: Valorization of cocoa pod side streams improves nutritional and sustainability aspects of chocolate
Source: Nat Food. 2024 May 21;5(5):423–32. doi: 10.1038/s43016-024-00967-2 (PMC11132982; doi:10.1038/s43016-024-00967-2)

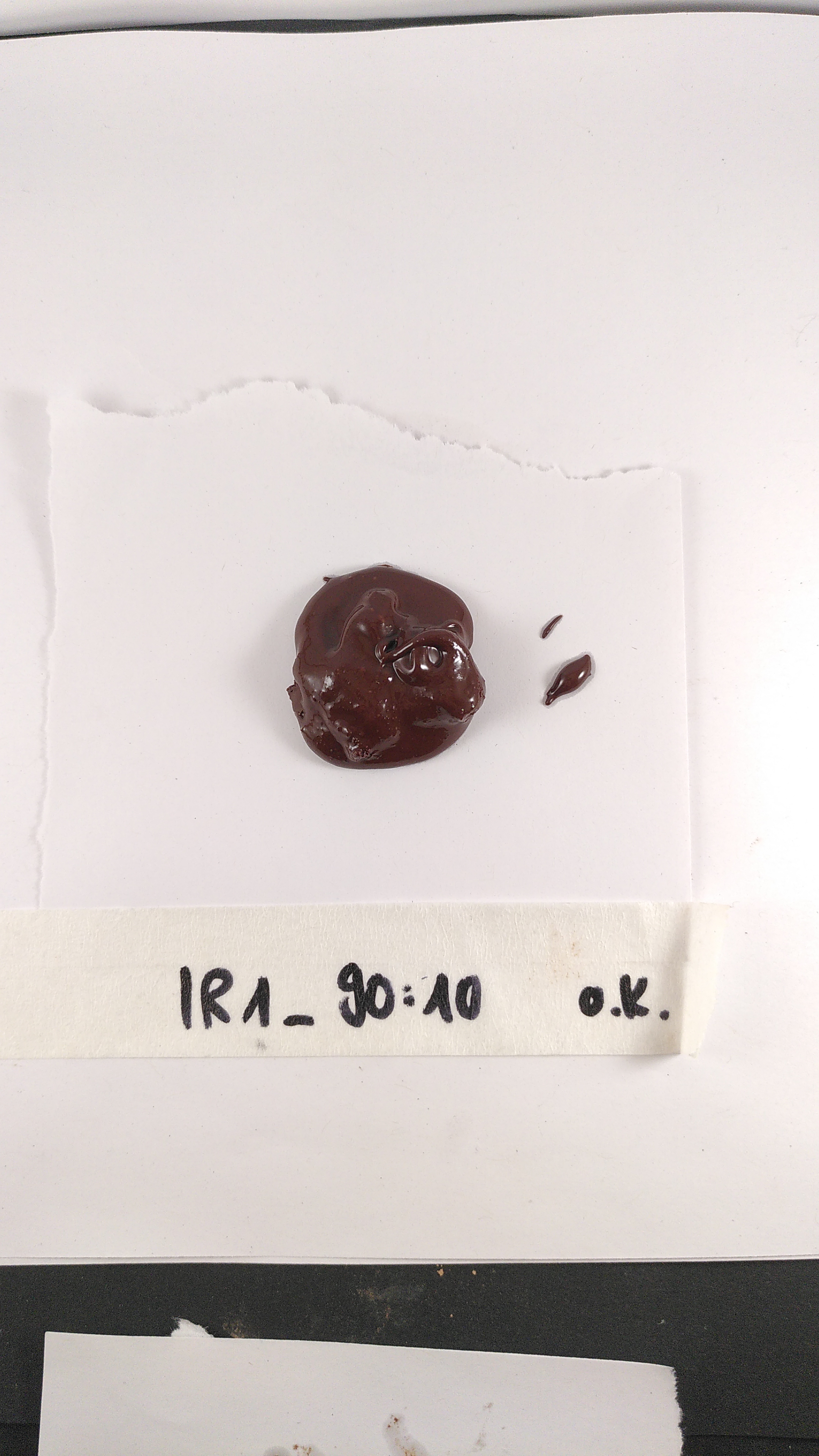

Supplement: Supplementary file 4 — Original, uncropped images of the simplified chocolate formulations displayed in Fig. 3. Grouped according to ECP concentration in gel. [file 43016_2024_967_MOESM4_ESM.zip › Fig3/10wtECP/ECP10GEL10.jpg]

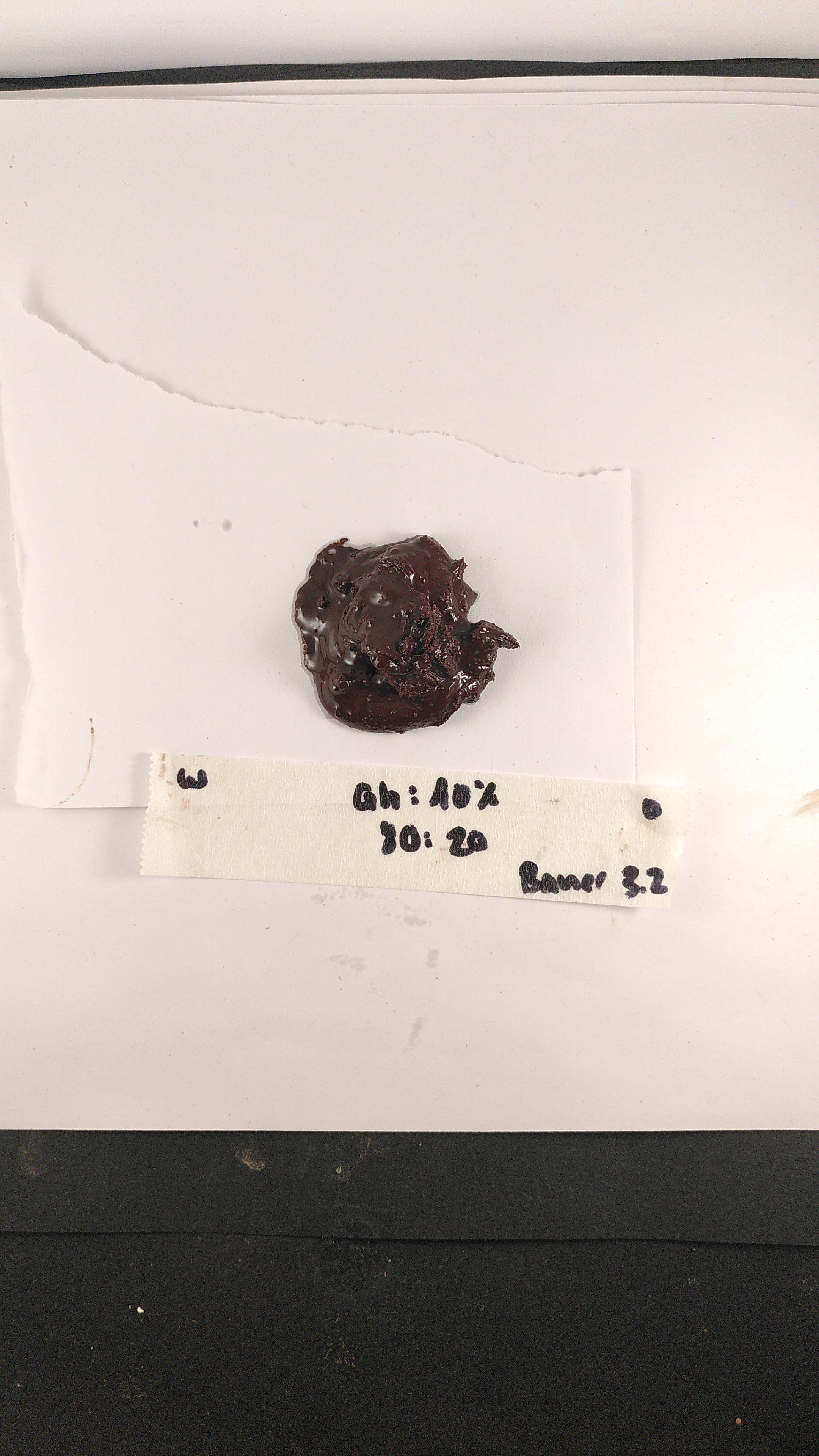

Supplement: Supplementary file 4 — Original, uncropped images of the simplified chocolate formulations displayed in Fig. 3. Grouped according to ECP concentration in gel. [file 43016_2024_967_MOESM4_ESM.zip › Fig3/10wtECP/ECP10GEL20.jpg]

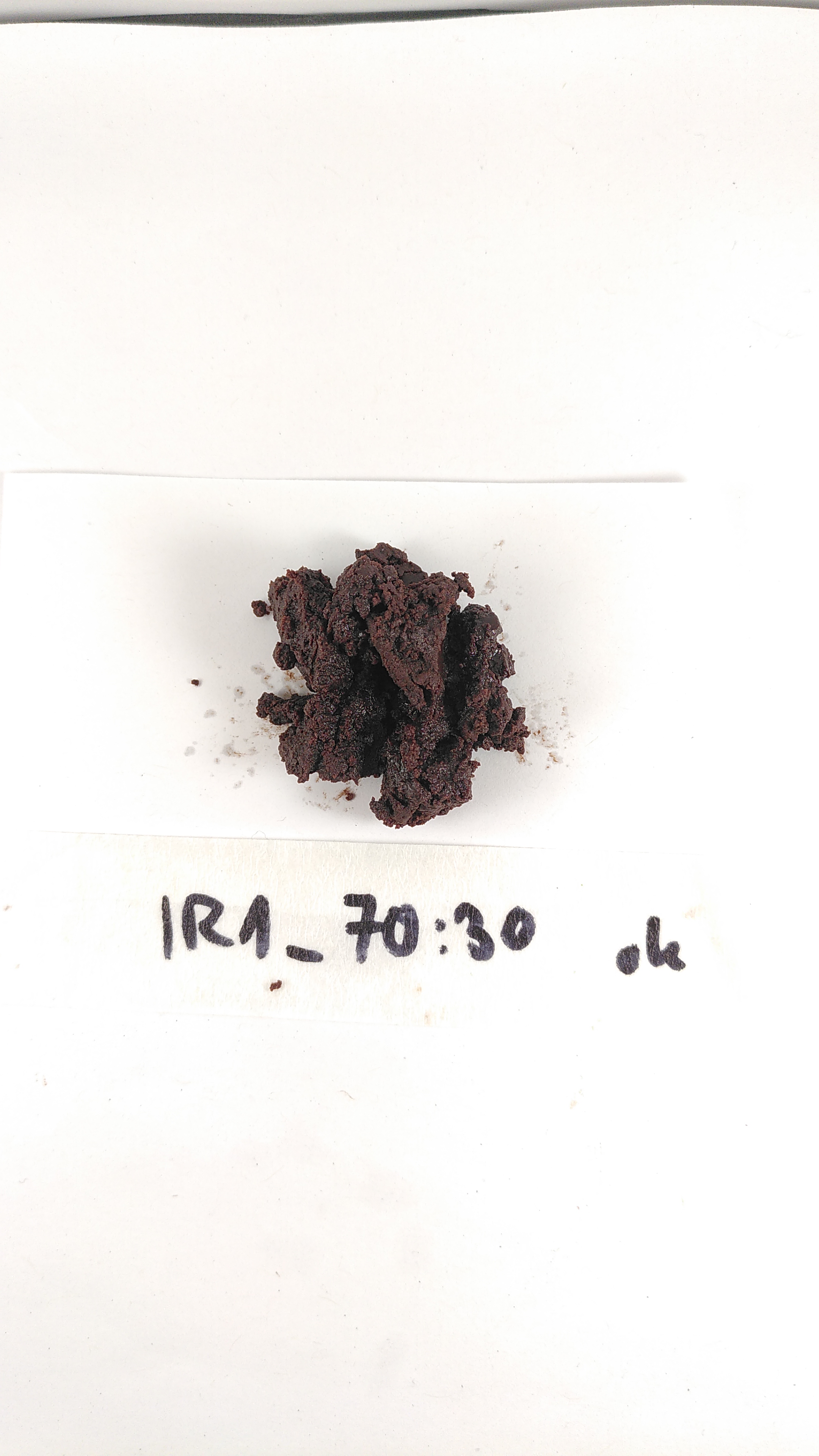

Supplement: Supplementary file 4 — Original, uncropped images of the simplified chocolate formulations displayed in Fig. 3. Grouped according to ECP concentration in gel. [file 43016_2024_967_MOESM4_ESM.zip › Fig3/10wtECP/ECP10GEL30.jpg]

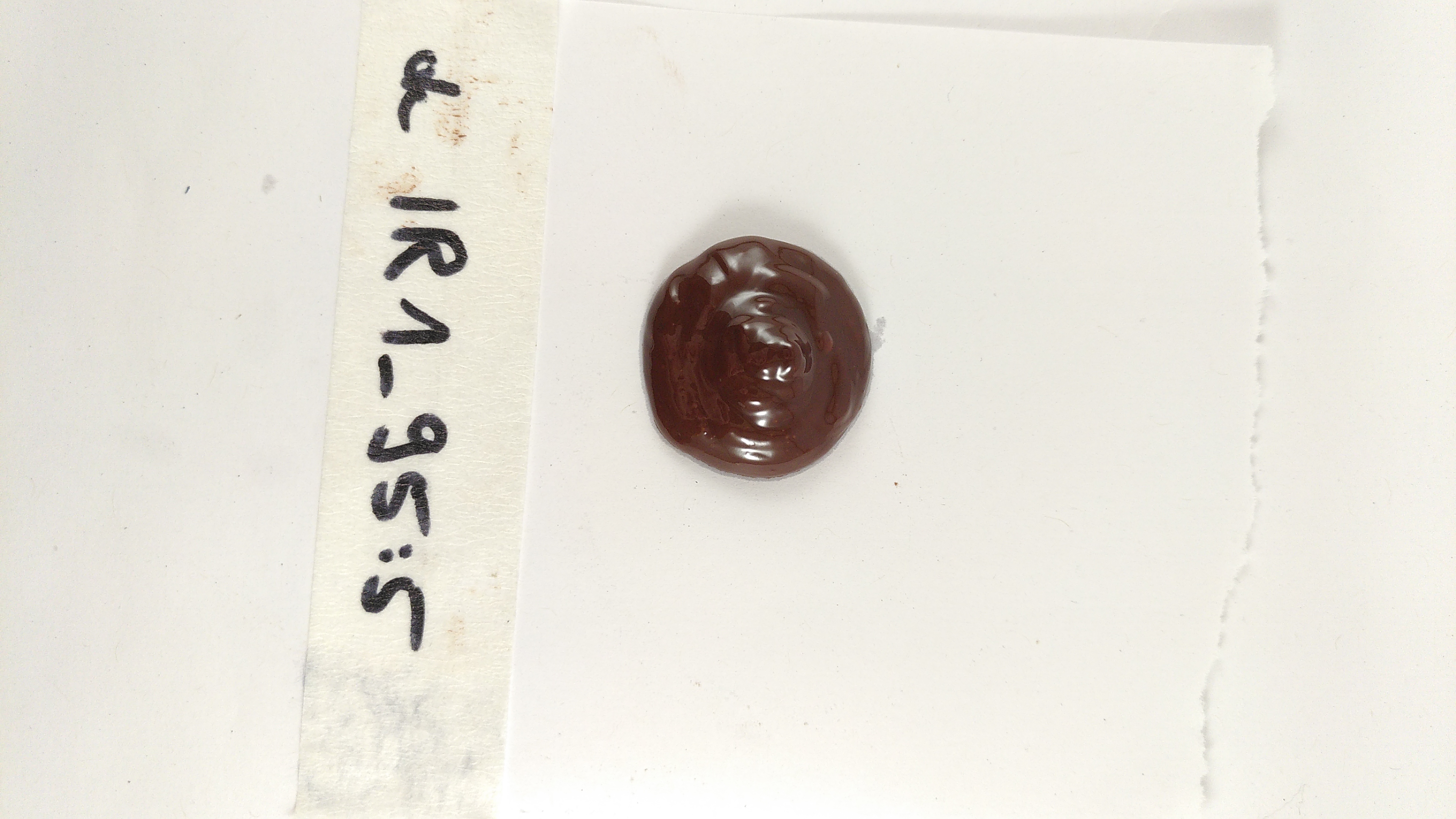

Supplement: Supplementary file 4 — Original, uncropped images of the simplified chocolate formulations displayed in Fig. 3. Grouped according to ECP concentration in gel. [file 43016_2024_967_MOESM4_ESM.zip › Fig3/10wtECP/ECP10GEL5.jpg]

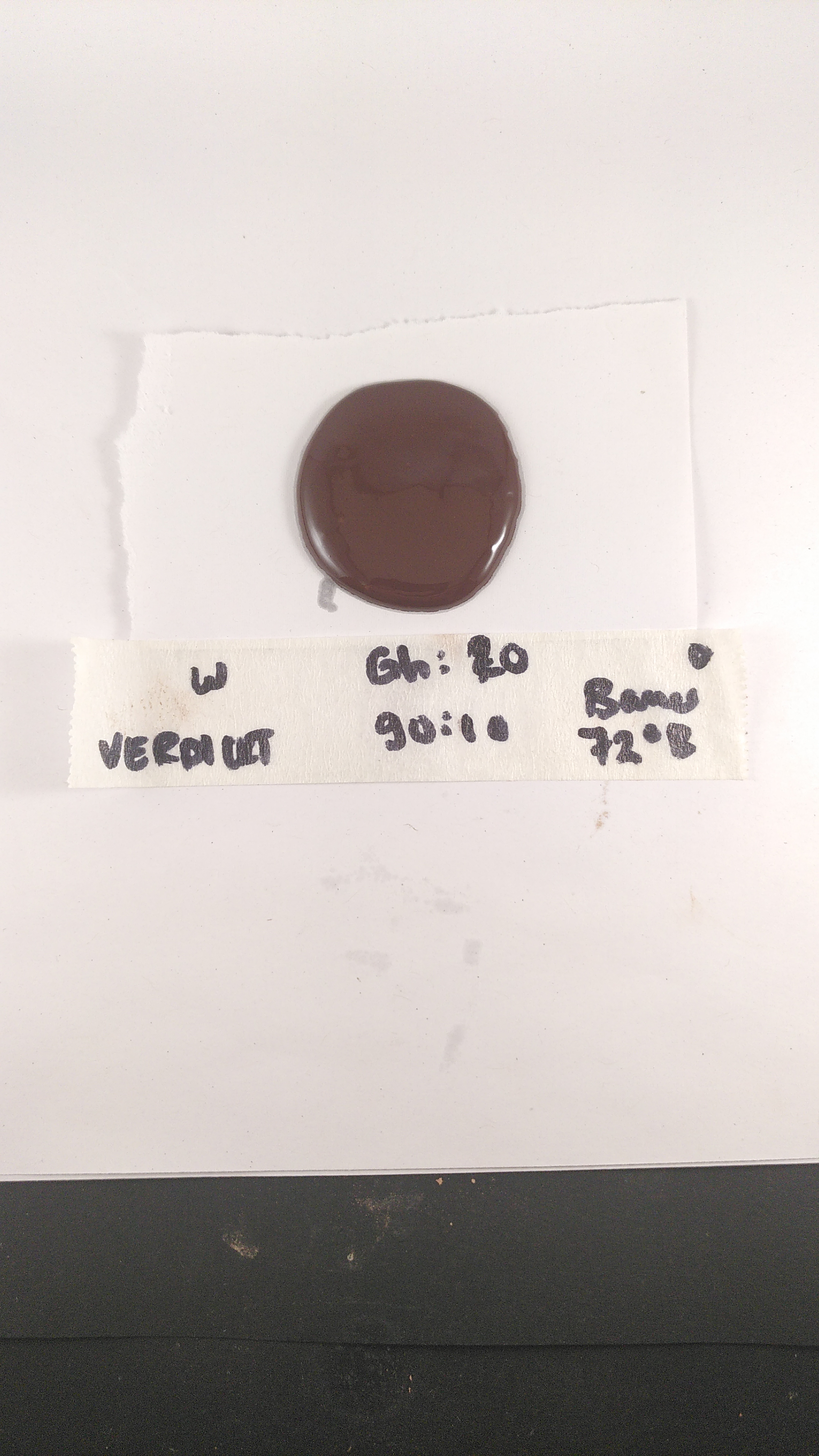

Supplement: Supplementary file 4 — Original, uncropped images of the simplified chocolate formulations displayed in Fig. 3. Grouped according to ECP concentration in gel. [file 43016_2024_967_MOESM4_ESM.zip › Fig3/20wtECP/ECP20GEL10.jpg]

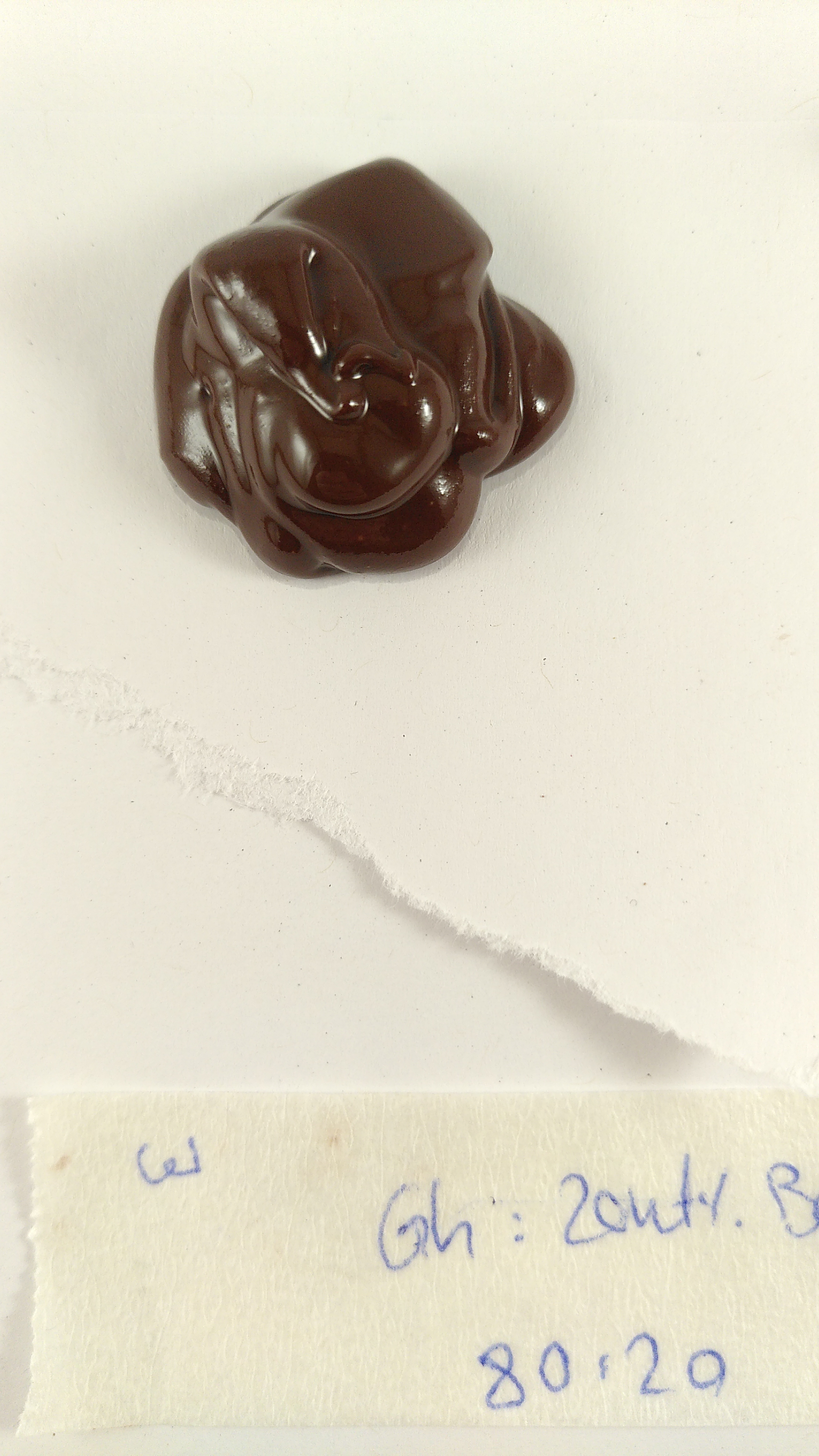

Supplement: Supplementary file 4 — Original, uncropped images of the simplified chocolate formulations displayed in Fig. 3. Grouped according to ECP concentration in gel. [file 43016_2024_967_MOESM4_ESM.zip › Fig3/20wtECP/ECP20GEL20.jpg]

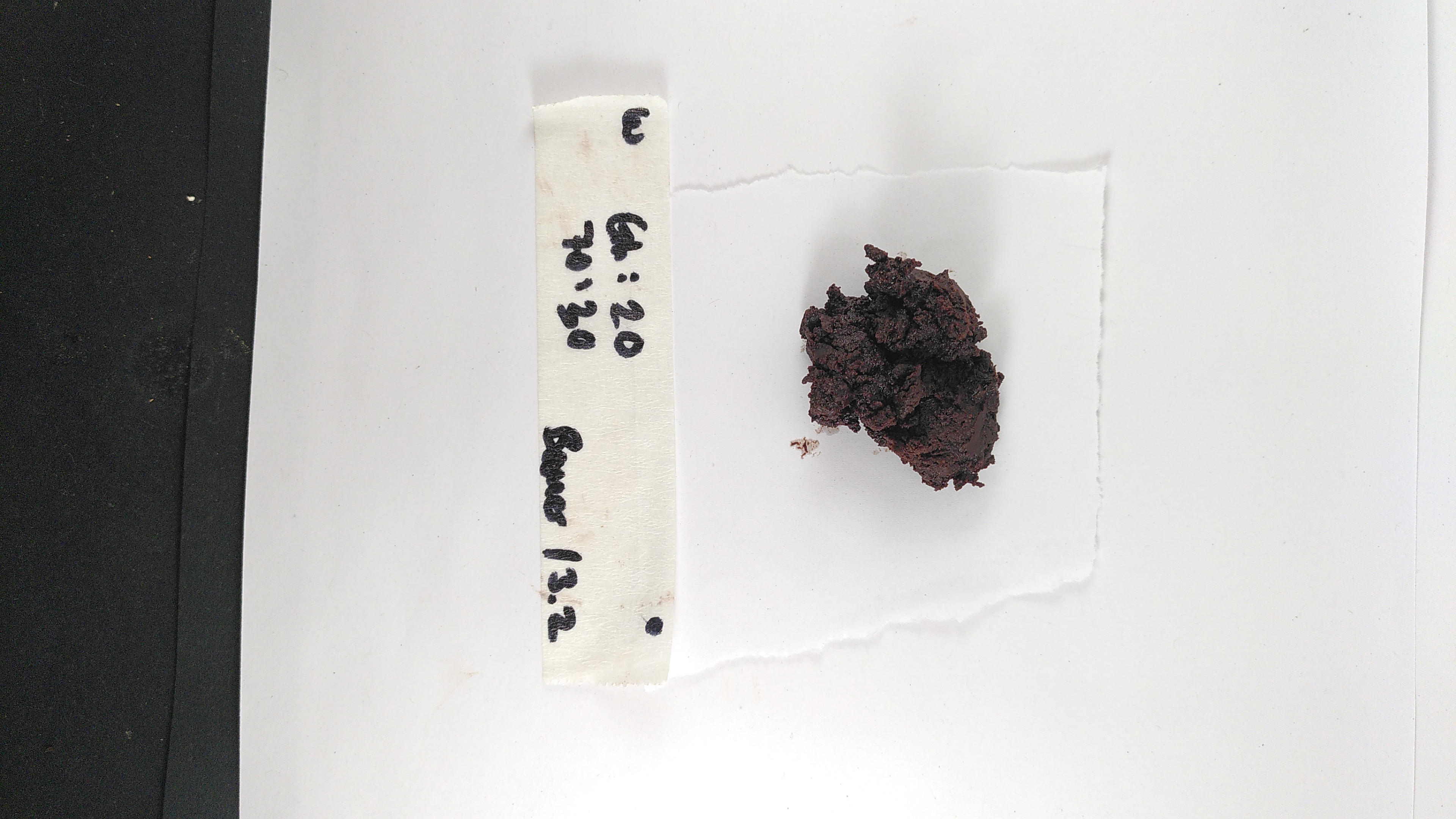

Supplement: Supplementary file 4 — Original, uncropped images of the simplified chocolate formulations displayed in Fig. 3. Grouped according to ECP concentration in gel. [file 43016_2024_967_MOESM4_ESM.zip › Fig3/20wtECP/ECP20GEL30.jpg]

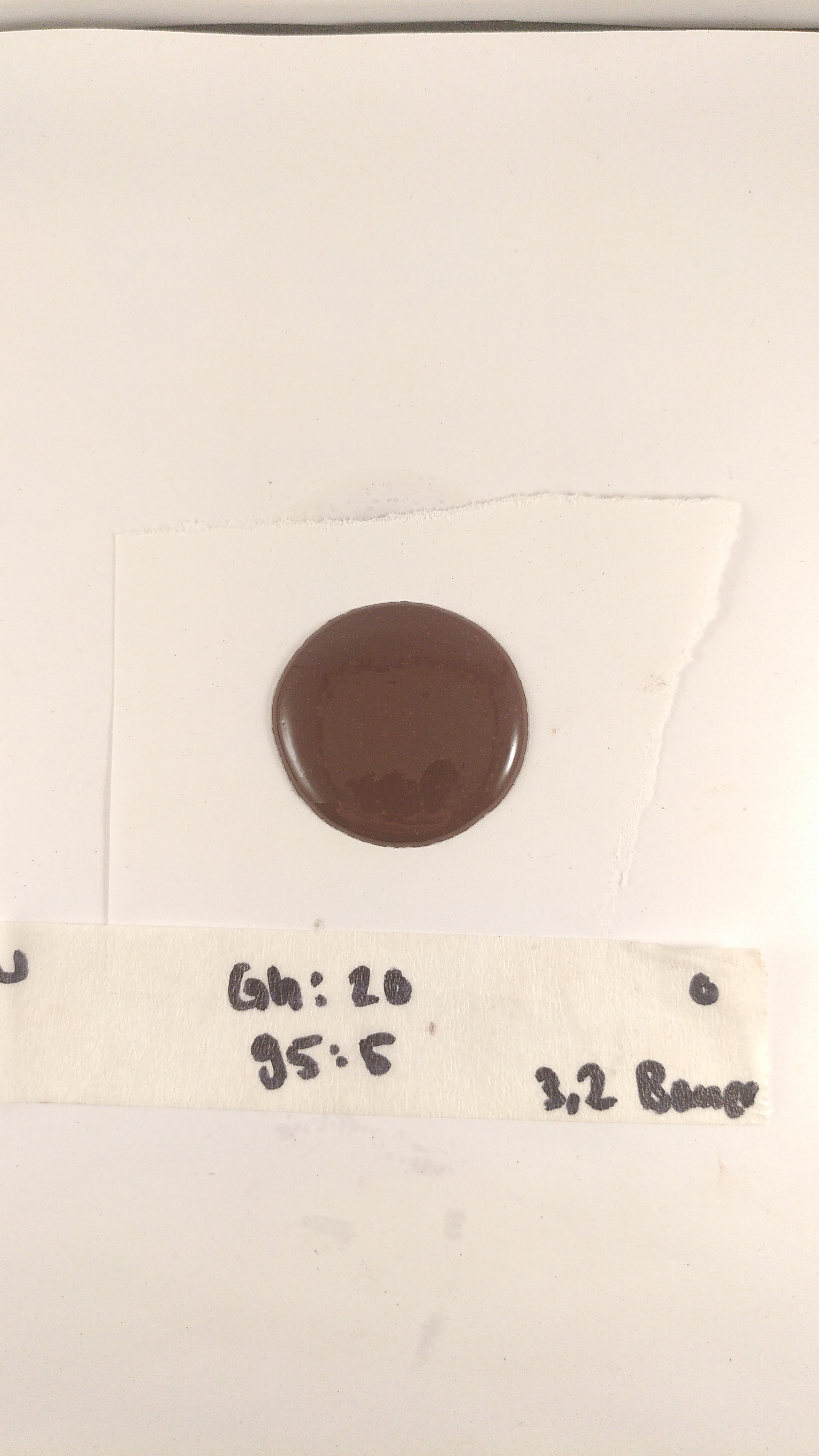

Supplement: Supplementary file 4 — Original, uncropped images of the simplified chocolate formulations displayed in Fig. 3. Grouped according to ECP concentration in gel. [file 43016_2024_967_MOESM4_ESM.zip › Fig3/20wtECP/ECP20GEL5.jpg]

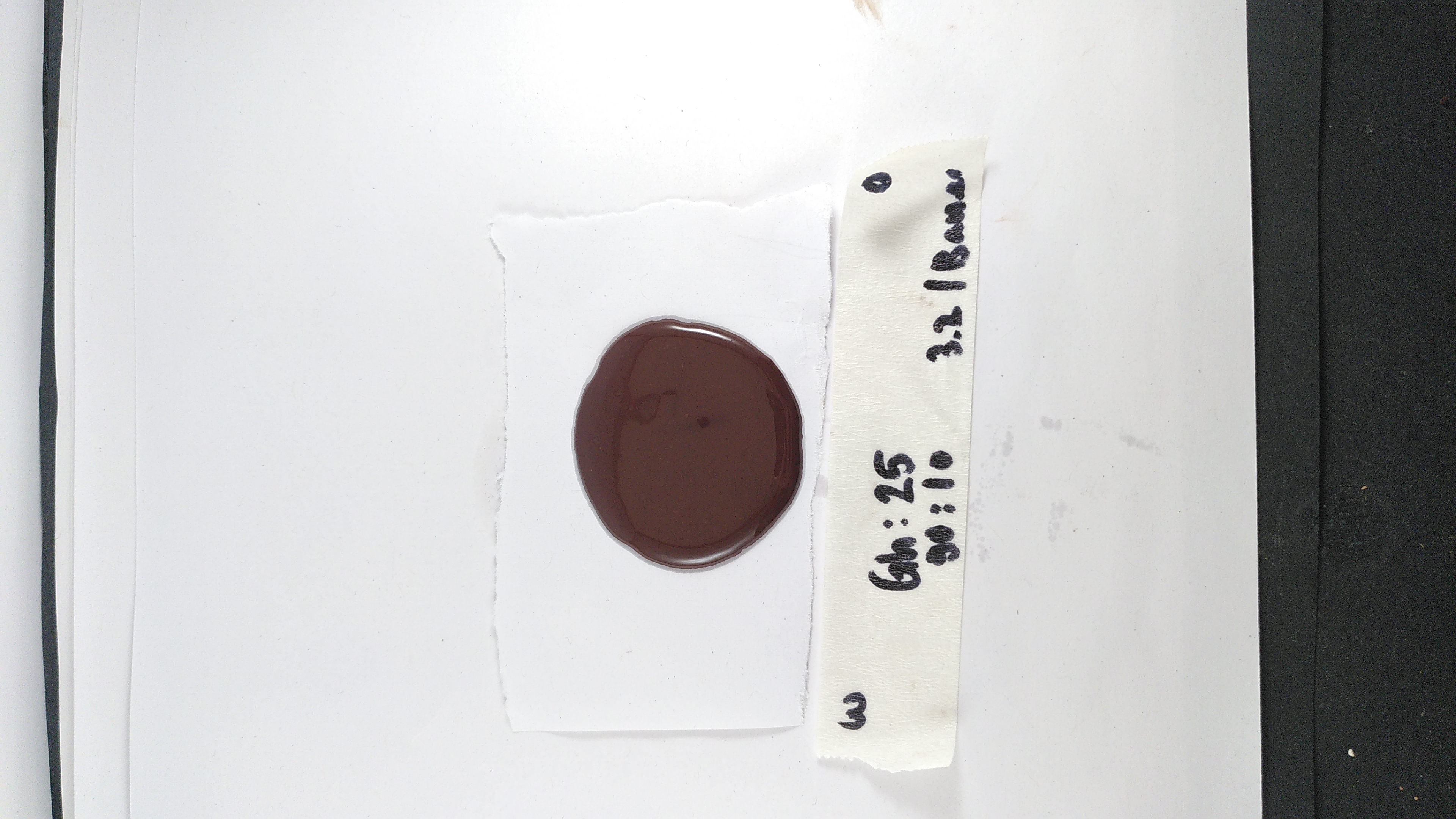

Supplement: Supplementary file 4 — Original, uncropped images of the simplified chocolate formulations displayed in Fig. 3. Grouped according to ECP concentration in gel. [file 43016_2024_967_MOESM4_ESM.zip › Fig3/25wtECP/ECP25GEL10.jpg]

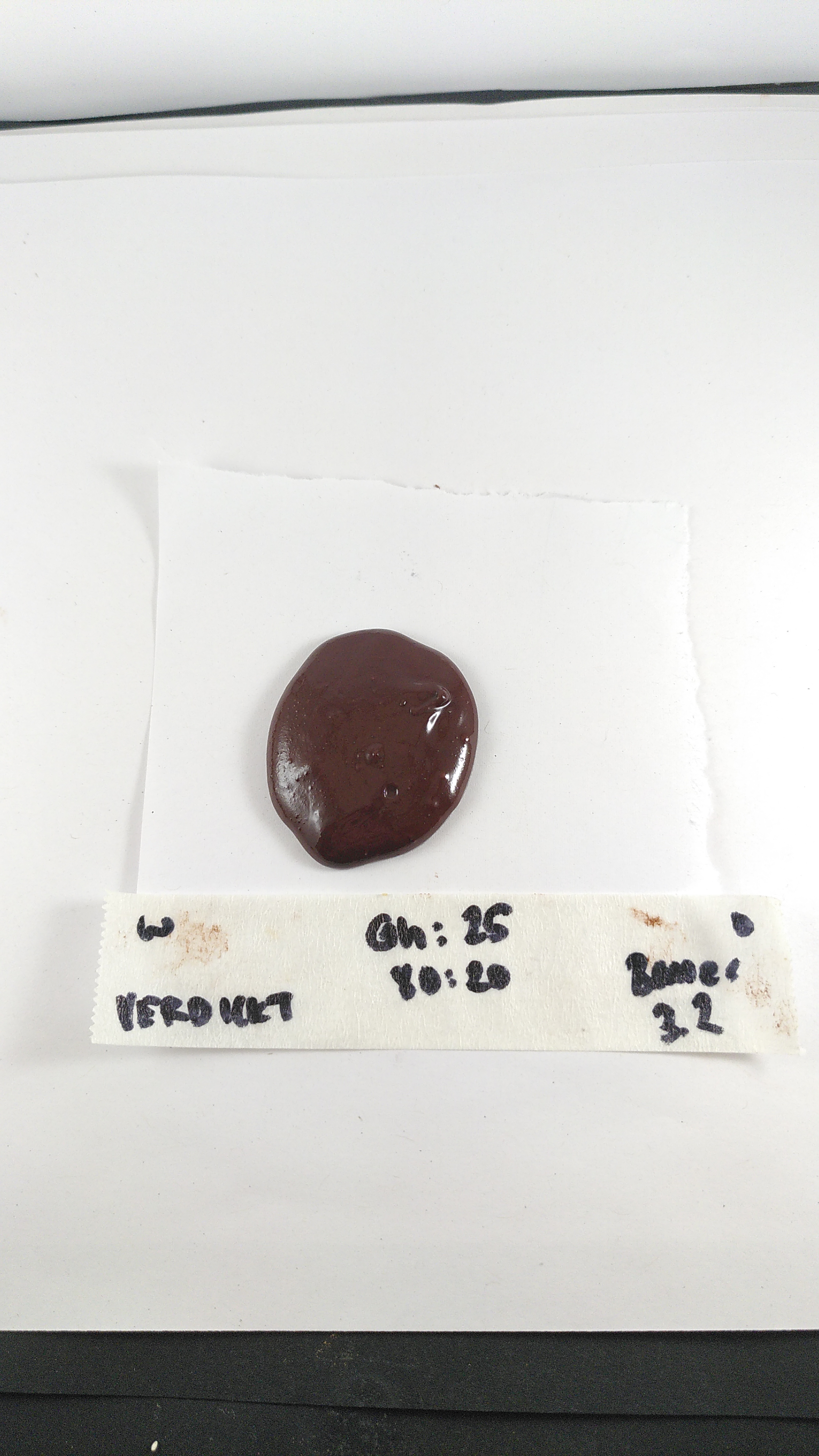

Supplement: Supplementary file 4 — Original, uncropped images of the simplified chocolate formulations displayed in Fig. 3. Grouped according to ECP concentration in gel. [file 43016_2024_967_MOESM4_ESM.zip › Fig3/25wtECP/ECP25GEL20.jpg]

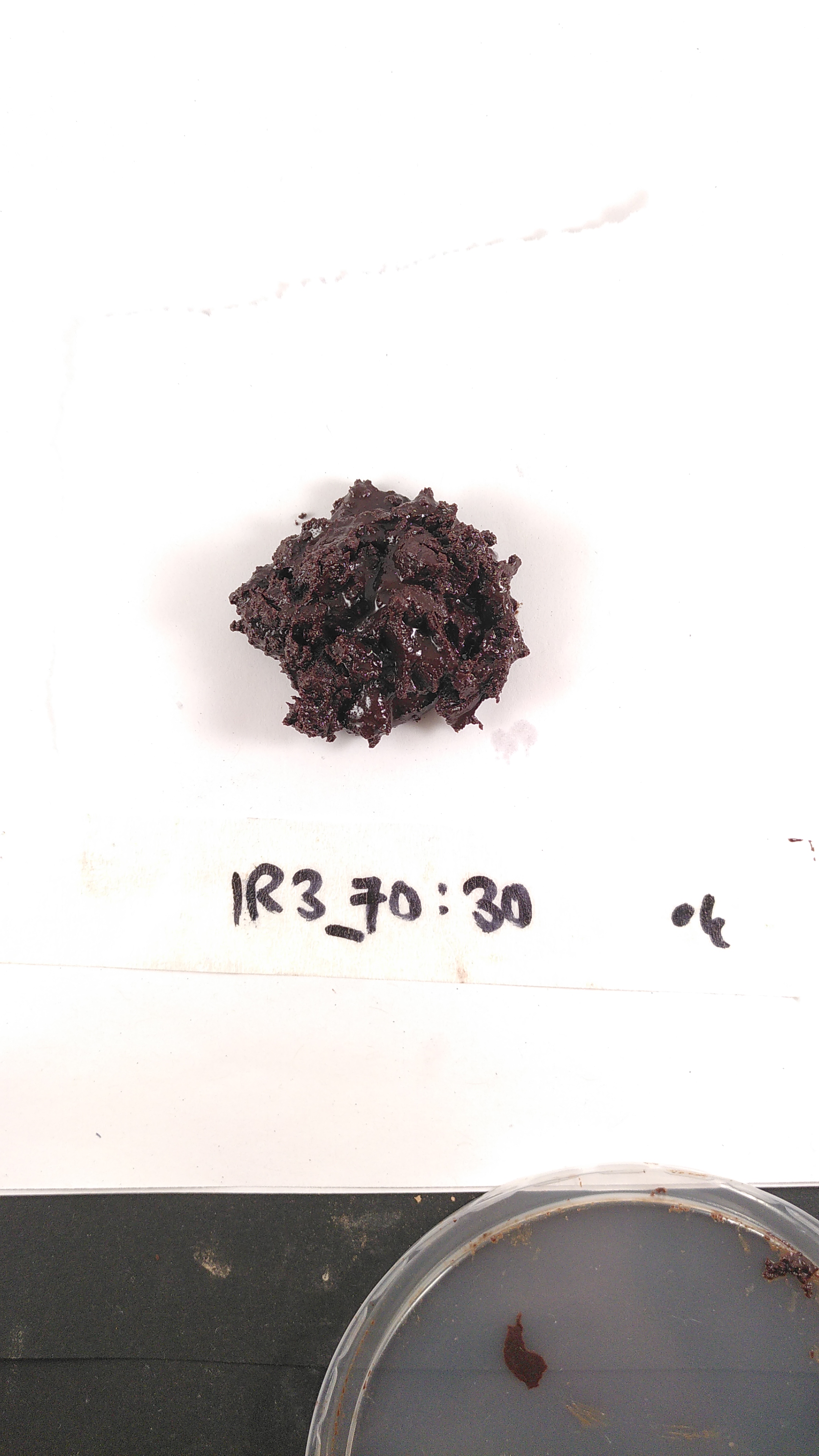

Supplement: Supplementary file 4 — Original, uncropped images of the simplified chocolate formulations displayed in Fig. 3. Grouped according to ECP concentration in gel. [file 43016_2024_967_MOESM4_ESM.zip › Fig3/25wtECP/ECP25GEL30.jpg]

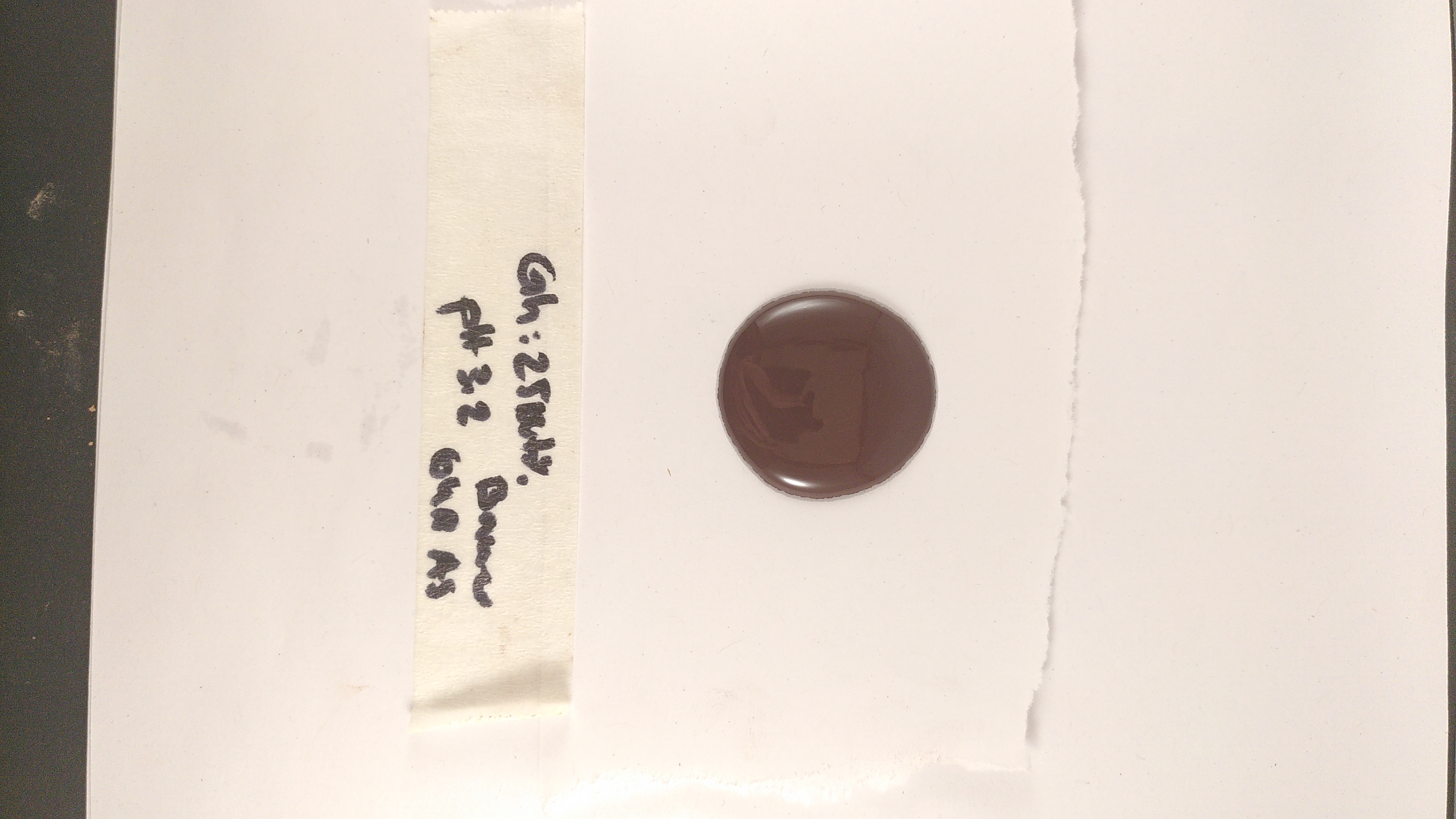

Supplement: Supplementary file 4 — Original, uncropped images of the simplified chocolate formulations displayed in Fig. 3. Grouped according to ECP concentration in gel. [file 43016_2024_967_MOESM4_ESM.zip › Fig3/25wtECP/ECP25GEL5.jpg]

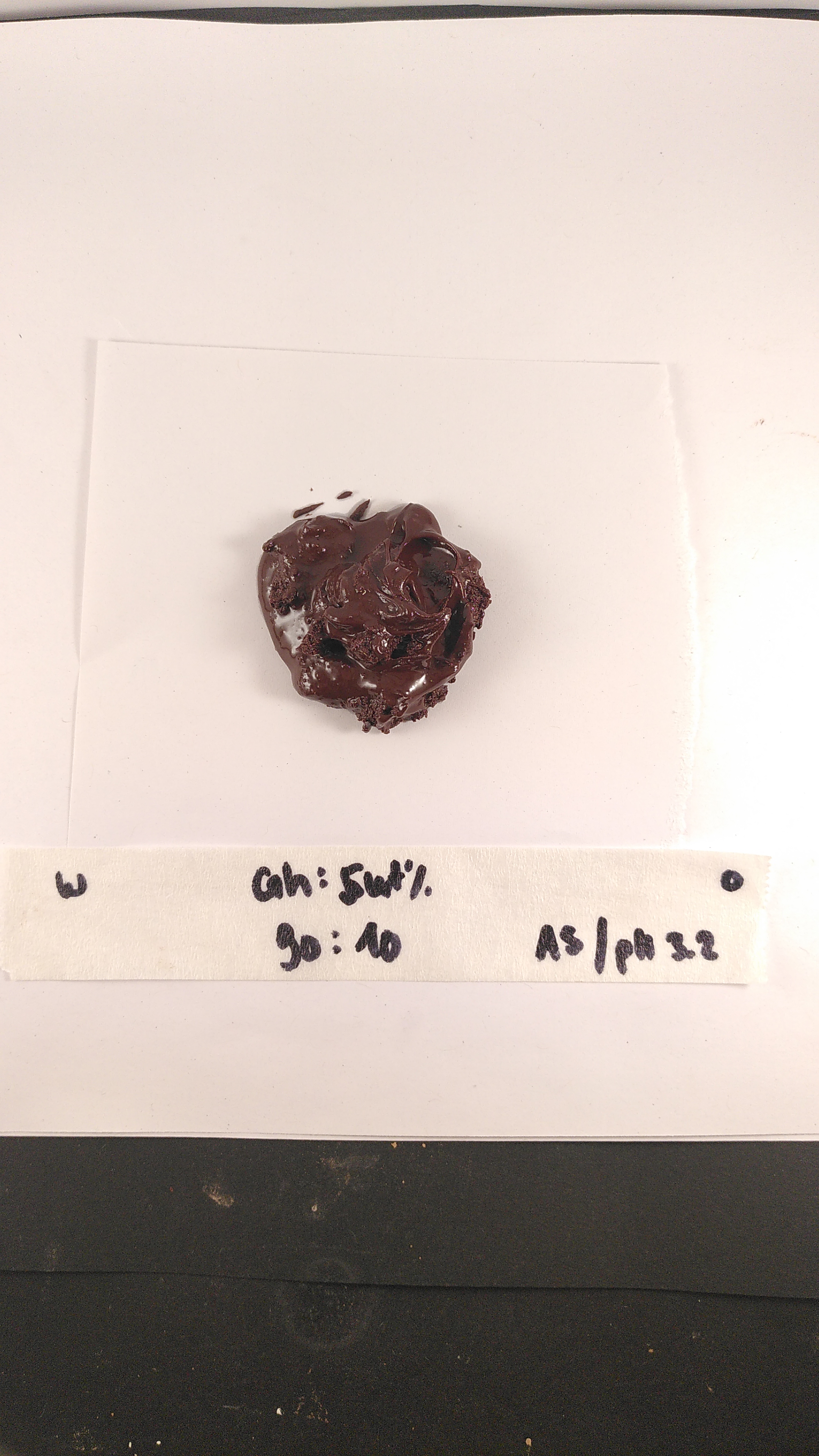

Supplement: Supplementary file 4 — Original, uncropped images of the simplified chocolate formulations displayed in Fig. 3. Grouped according to ECP concentration in gel. [file 43016_2024_967_MOESM4_ESM.zip › Fig3/5wtECP/ECP5GEL10.jpg]

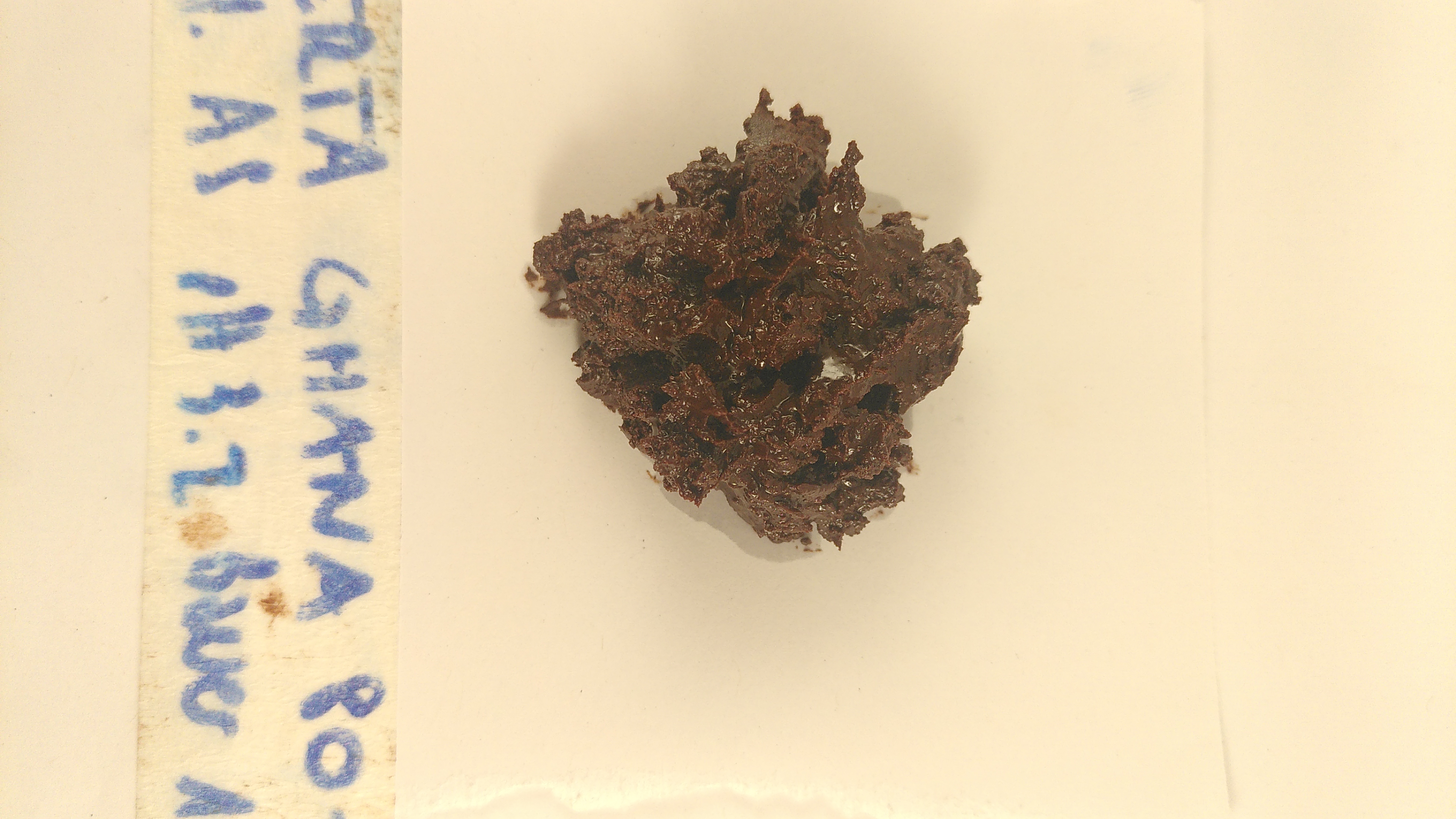

Supplement: Supplementary file 4 — Original, uncropped images of the simplified chocolate formulations displayed in Fig. 3. Grouped according to ECP concentration in gel. [file 43016_2024_967_MOESM4_ESM.zip › Fig3/5wtECP/ECP5GEL20.jpg]

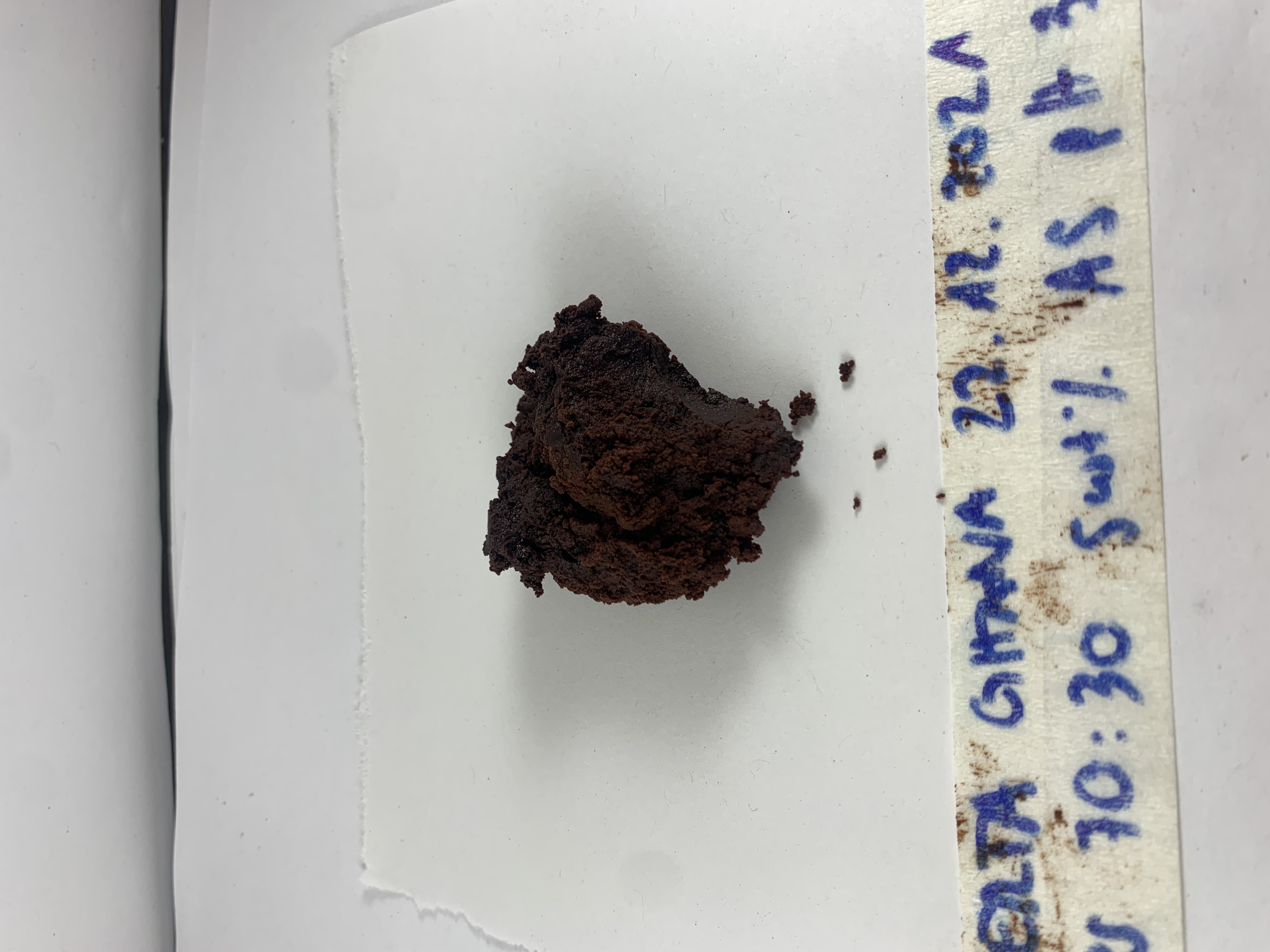

Supplement: Supplementary file 4 — Original, uncropped images of the simplified chocolate formulations displayed in Fig. 3. Grouped according to ECP concentration in gel. [file 43016_2024_967_MOESM4_ESM.zip › Fig3/5wtECP/ECP5GEL30.JPG]

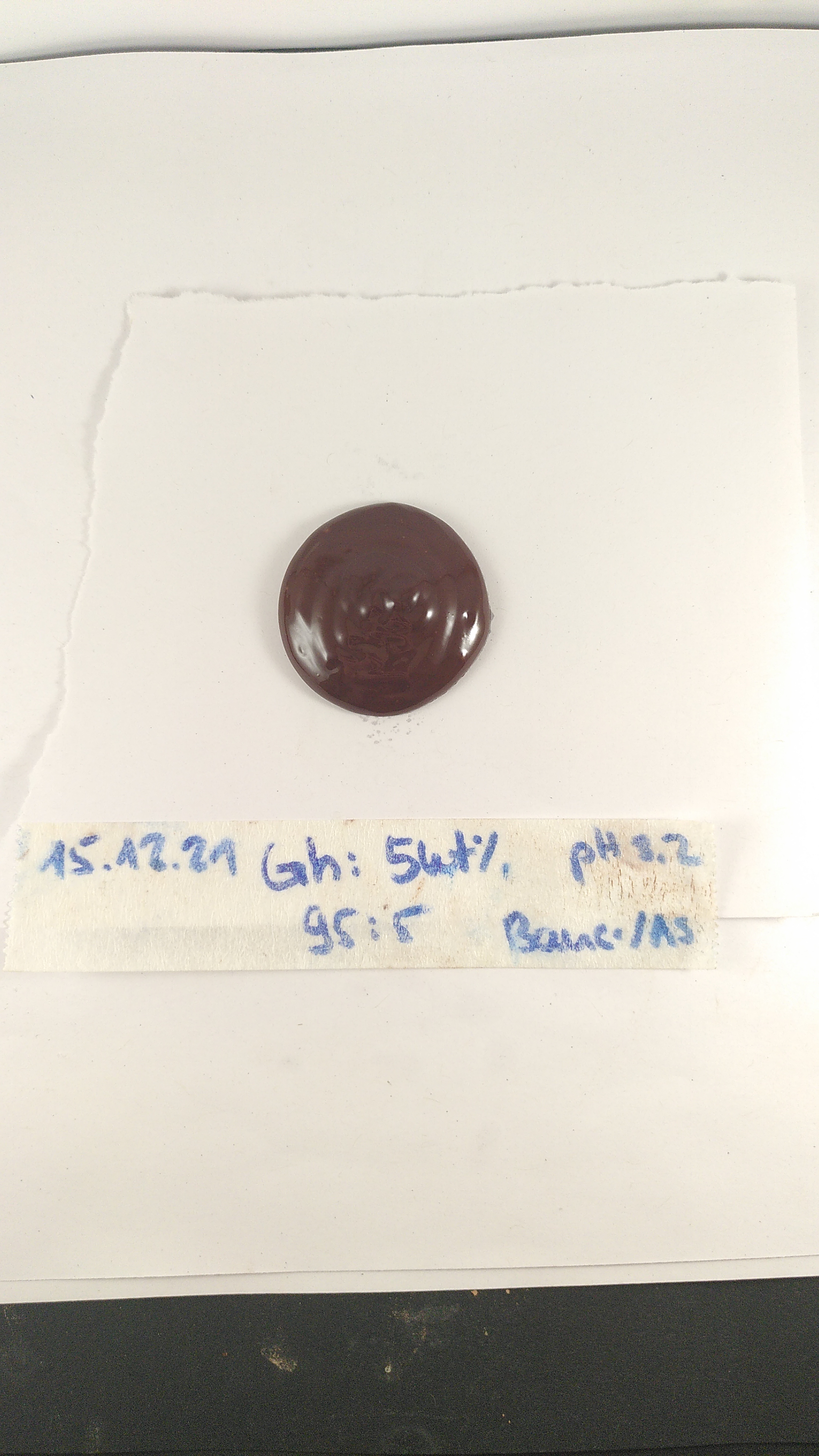

Supplement: Supplementary file 4 — Original, uncropped images of the simplified chocolate formulations displayed in Fig. 3. Grouped according to ECP concentration in gel. [file 43016_2024_967_MOESM4_ESM.zip › Fig3/5wtECP/ECP5GEL5.jpg]
